# MicroRNA miR124 is required for the expression of homeostatic synaptic plasticity

**DOI:** 10.1038/ncomms10045

**Published:** 2015-12-01

**Authors:** Qingming Hou, Hongyu Ruan, James Gilbert, Guan Wang, Qi Ma, Wei-Dong Yao, Heng-Ye Man

**Affiliations:** 1 Department of Biology, Boston University, 5 Cummington Mall, Boston, Massachusetts 02215, USA; 2 Division of Neuroscience, New England Primate Research Center, Harvard Medical School, Southborough, Massachusetts 01772, USA; 3 Department of Pharmacology and Experimental Therapeutics, Boston University, 5 Cummington Mall, Boston, Massachusetts 02215, USA

## Abstract

Homeostatic synaptic plasticity is a compensatory response to alterations in neuronal activity. Chronic deprivation of neuronal activity results in an increase in synaptic AMPA receptors (AMPARs) and postsynaptic currents. The biogenesis of GluA2-lacking, calcium-permeable AMPARs (CP-AMPARs) plays a crucial role in the homeostatic response; however, the mechanisms leading to CP-AMPAR formation remain unclear. Here we show that the microRNA, miR124, is required for the generation of CP-AMPARs and homeostatic plasticity. miR124 suppresses GluA2 expression via targeting its 3′-UTR, leading to the formation of CP-AMPARs. Blockade of miR124 function abolishes the homeostatic response, whereas miR124 overexpression leads to earlier induction of homeostatic plasticity. miR124 transcription is controlled by an inhibitory transcription factor EVI1, acting by association with the deacetylase HDAC1. Our data support a cellular cascade in which inactivity relieves EVI1/HDAC-mediated inhibition of miR124 gene transcription, resulting in enhanced miR124 expression, formation of CP-AMPARs and subsequent induction of homeostatic synaptic plasticity.

## Abstract (web summary)

GluA2-lacking AMPA receptors are known to play a role in homeostatic plasticity. Here, the authors show that spiking activity blockade disinhibits mir124 transcription, which in turn suppresses GluA2 mRNA translation, thereby contributing to synaptic upscaling in hippocampal cells.

## Graphical abstract

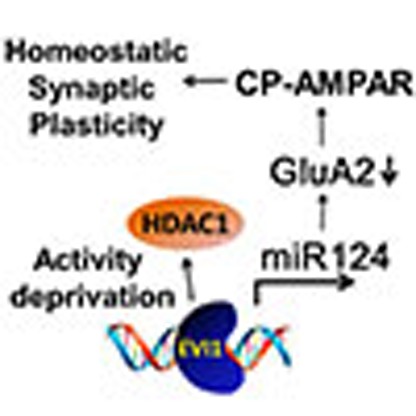

Homeostatic plasticity is a compensatory regulation in neuronal activity, which is crucial for the maintenance of neuronal and neural circuit stability^1^,^2^,^3^,^4^,^5^. A major cellular mechanism underlying the homeostatic regulation is to adjust synaptic strength in a negative feedback manner, that is, homeostatic synaptic plasticity (HSP)^2^,^3^,^6^. Studies have shown that in neuronal cultures chronic suppression of cell activity results in an increase in the amount of synaptic AMPARs and therefore strengthened synaptic transmission^7^,^8^,^9^. Similar regulation has also been observed *in vivo*^10^,^11^,^12^,^13^,^14^,^15^,^16^. However, the initial molecular steps and signaling cascades that lead to the expression of HSP remain less well understood.

Most AMPAR heterotetrameric complexes contain GluA2 subunits and are permeable only to sodium and potassium. When constituted without GluA2, AMPARs become permeable to both sodium and calcium. An accumulating amount of work has established an important role for CP-AMPARs in HSP. Following activity deprivation, the level of GluA1 expression is preferentially increased over GluA2 (refs 17, 18, 19), suggesting the formation of GluA2-lacking AMPARs. Consistently, under activity deprivation, AMPAR-mediated currents show inward rectification and become sensitive to CP-AMPAR-selective antagonists philanthotoxin-433 (PhTx) or Naspm^10^,^17^,^18^,^19^,^20^,^21^. Interestingly, multiple signaling molecules involved in HSP, including TNF-α, retinoic acid, Arc/Arg3.1 and integrin β3, can cause an imbalance in GluA1 and GluA2 expression, and thus biogenesis of CP-AMPARs^18^,^22^,^23^. Our own study also shows that synaptic homeostatic regulation requires the activity of CP-AMPARs. Application of PhTx during activity deprivation abolishes the expression of AMPAR-mediated homeostatic regulation^21^. Interestingly, the blockade of homeostatic plasticity is observed only when PhTx is applied at the early stage of activity deprivation, but not the later phase^21^, indicating that CP-AMPARs function as a signaling cue for the induction of homeostatic synaptic regulation. However, how the GluA2-lacking CP-AMPARs are generated by inactivity remains unknown.

MicroRNAs (miRNAs) are 19–25 (∼22) noncoding nucleotide RNAs that play important roles in the posttranscriptional regulation of gene expression^24^,^25^,^26^,^27^. miRNAs repress translation by binding to specific complementary sequences located in the 3′-untranslated region (3′-UTR) of target messenger RNAs (mRNAs). The miRNA–mRNA interaction normally requires six to eight base pairs of perfect complementarity between the miRNA 5′ terminus (seed sequence) and a cognate miRNA target site in the mRNA 3′-UTR^26^,^28^. At the posttranscriptional level, miRNA can regulate gene expression in a tight spatial and temporal manner. There are more than 1,000 miRNAs in humans, and most of them are highly conserved across species. Some have a general expression pattern, others are specifically expressed in certain tissues or cell types, and expression can be spatially and temporally restricted.

miR124 is one of the most abundant miRNAs expressed in the brain, accounting for more than a quarter of all brain miRs^29^,^30^. There are three independent loci of miR124 in vertebrates and all produce the same mature miR124 (ref. 31). miR124 is expressed in neurons, but not astrocytes, and the levels of miR124 increase over time in the developing brain^30^,^32^,^33^ In cultured cortical neurons, we also found that miR124 expression is continuously increased after plating. miR124 has been shown to play a key role in neuronal differentiation and neurogenesis^34^. Overexpression of miR124 promotes differentiation of precursor cells into a neuronal phenotype and stimulates neurite growth^35^. When functional miR124 is increased in mouse or human cells *in vitro*, the global mRNA expression profile shifts toward neuronal mRNA population^36^,^37^. Deletion of miR124 can lead to major developmental phenotypes including decreased brain size, defective axonal outgrowth and cell death^38^. Several genes involved in neurogenesis have been identified as miR124 targets, including SCP1, BAF53a, Ptbp1, RhoG, Jagged1 and Sox9. However, the role of miR124 in the regulation of neurotransmitter receptors and synaptic plasticity remains largely unknown^39^.

We explored the role of miR124 in AMPAR subunit expression and in homeostatic plasticity. We find that miR124, via interaction with the 3′-UTR of the GluA2, but not GluA1 subunit, causes a selective reduction in GluA2 levels and the formation of CP-AMPARs. The expression of miR124 is activity dependent. Suppression of neuronal activity induces an increase in miR124 expression, consistent with a role for miR124 in the homeostatic response during activity deprivation. Blockade of miR124 function completely abolishes inactivity-induced homeostatic plasticity. In addition, we confirm that in neurons, miR124 transcription is controlled by an inhibitory transcription factor EVI1 via its association with a histone deacetylase, HDAC1. Interestingly, miR124 alone is not sufficient to induce a homeostatic response; rather, it is able to accelerate the induction of homeostatic plasticity under the condition of activity deprivation.

## Results

### miR124 specifically targets AMPAR subunit GluA2

**Figure 1:**
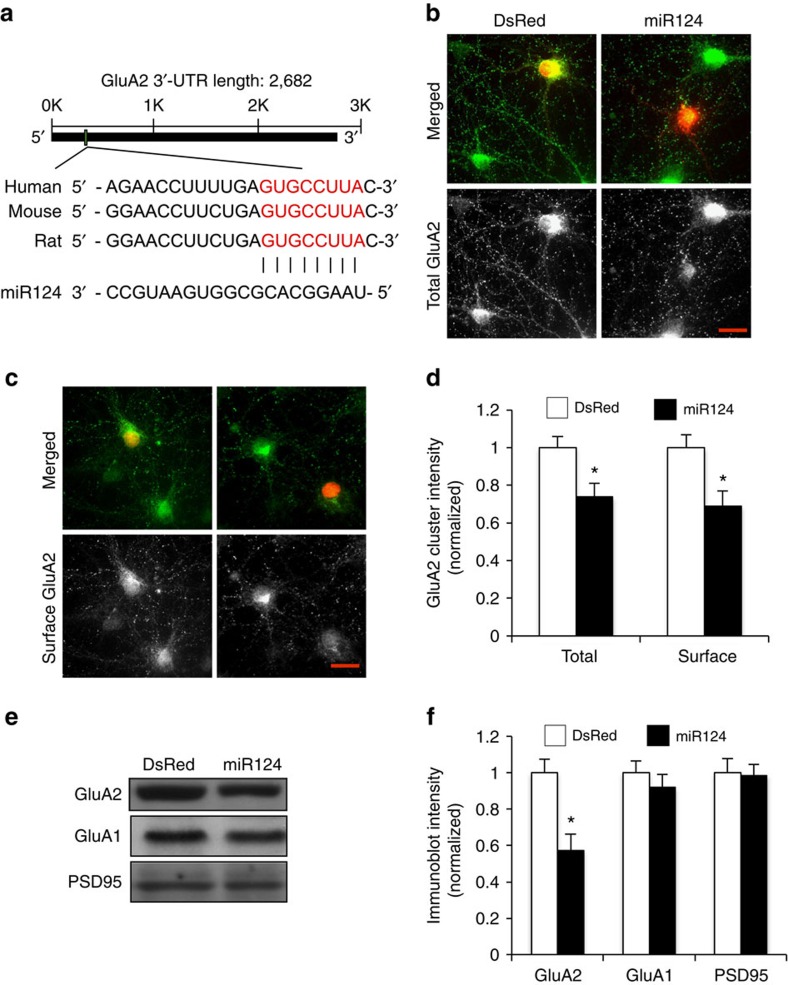
miR124 expression selectively suppresses GluA2 expression. (**a**) The binding site for the miR124 seed sequence in GluA2 mRNA 3′-UTR is highly conserved in human, mouse and rat. (**b**–**d**) Cultured hippocampal neurons were transfected with miR124 (containing DsRed) or DsRed at DIV12. Total and cell-surface GluA2 were immunostained (green) at DIV14 under permeant and non-permeant conditions, respectively. GluA2 puncta intensity was measured. Bar graphs represent mean±s.e., *P<0.05, *t*-test. Image scale bars=10 μm. (**e**,**f**) DIV12 cortical neurons were infected with lentiviral constructs of miR124 or DsRed control for 3 days, and cell lysates were probed for GluA1, GluA2 and PSD95. miR124 induced a decrease in the expression of GluA2, but not GluA1 or PSD95. Bar graphs represent mean±s.e., *n*=4 experiments, *P<0.05, *t*-test.

To examine whether the expression of AMPAR GluA2 subunits is subject to the regulation of microRNA, we used Targetscan software to search for potential miRNAs that target the 3′-UTR sequence of GluA2. A search of the database revealed several potential miRNAs, including miR124, mir30, mir181 and mir218. Among them, miR124 is the most abundant miRNA expressed in neurons^29^. miR124 binds to an 8-bp sequence in the GluA2 3′-UTR, while no targeting sites were detected in GluA1 3′-UTR. This binding site is localized at the 5′ end of the 3′-UTR (187–194 bp from the 5′ end), the optimal location for efficient microRNA function^25^. Furthermore, the miR124 binding sequence in the GluA2 3′-UTR is evolutionarily conserved (Fig. 1a). Therefore, miR124 was considered the best candidate for GluA2 regulation. Previous studies showed that miR124 is required for neuronal determination and differentiation but its roles in mature neurons remain unclear. We first wanted to know whether miR124 expression could cause gross changes in neuron morphology or cellular conditions. We transfected cultured hippocampal neurons at day *in vitro* (DIV) 11 with a plasmid containing DsRed and the miR124 sequence inserted in an intron of the *DsRed* gene. Regular DsRed construct was transfected in sister cultures as a control. No toxicity or obvious developmental and structural changes were observed in neurons 3 days after transfection. Dendritic arborization and synapse density as measured with PSD95 staining showed no difference compared with the transfected DsRed control cells (Supplementary Fig. 1).

GluA2 mRNA was predicted to be the target of miR124 by sequence matching. To validate this, we transfected miR124 in cultured rat hippocampal neurons at DIV 11. Two days after transfection, neurons were immunostained for AMPARs under permeant and non-permeant conditions for the analysis of total and surface receptors, respectively. When synaptic puncta intensity was measured, we found that miR124 overexpression resulted in a significant reduction in both total and surface GluA2 puncta compared with the DsRed control (Fig. 1b–d; total: 74±7% of control value, *P*<0.05, *n*=392 puncta from 14 cells, mean±s.e.; surface: 69±8% of control value, *P*<0.05, *n*=368 puncta from 12 cells; *t*-test). In contrast, puncta intensity of GluA1 showed no change (Supplementary Fig. 2, 102±10% of control value, *P*>0.05, *n*=485 puncta from 10 cells; *t*-test). To further confirm these findings in a more non-biased manner with biochemistry, we subcloned miR124 into a lentiviral vector and infected cultured cortical neurons with concentrated miR124 virus. In line with immunostaining data, western blots using infected cell lysates demonstrated a marked reduction in GluA2 protein levels, whereas no changes were found in the abundance of GluA1 and the synaptic scaffolding protein PSD95 (Fig. 1e,f, GluA2: 57.4±8.9% of control; GluA1: 92.1±7.2% of control; PSD95: 98.5±6.1% of control, *n*=4 each). These data indicate that miR124 selectively downregulates GluA2, and the effect is not a result of general suppression of protein synthesis or due to synapse removal.

### miR124 targets the 3′-UTR of GluA2 mRNA

**Figure 2:**
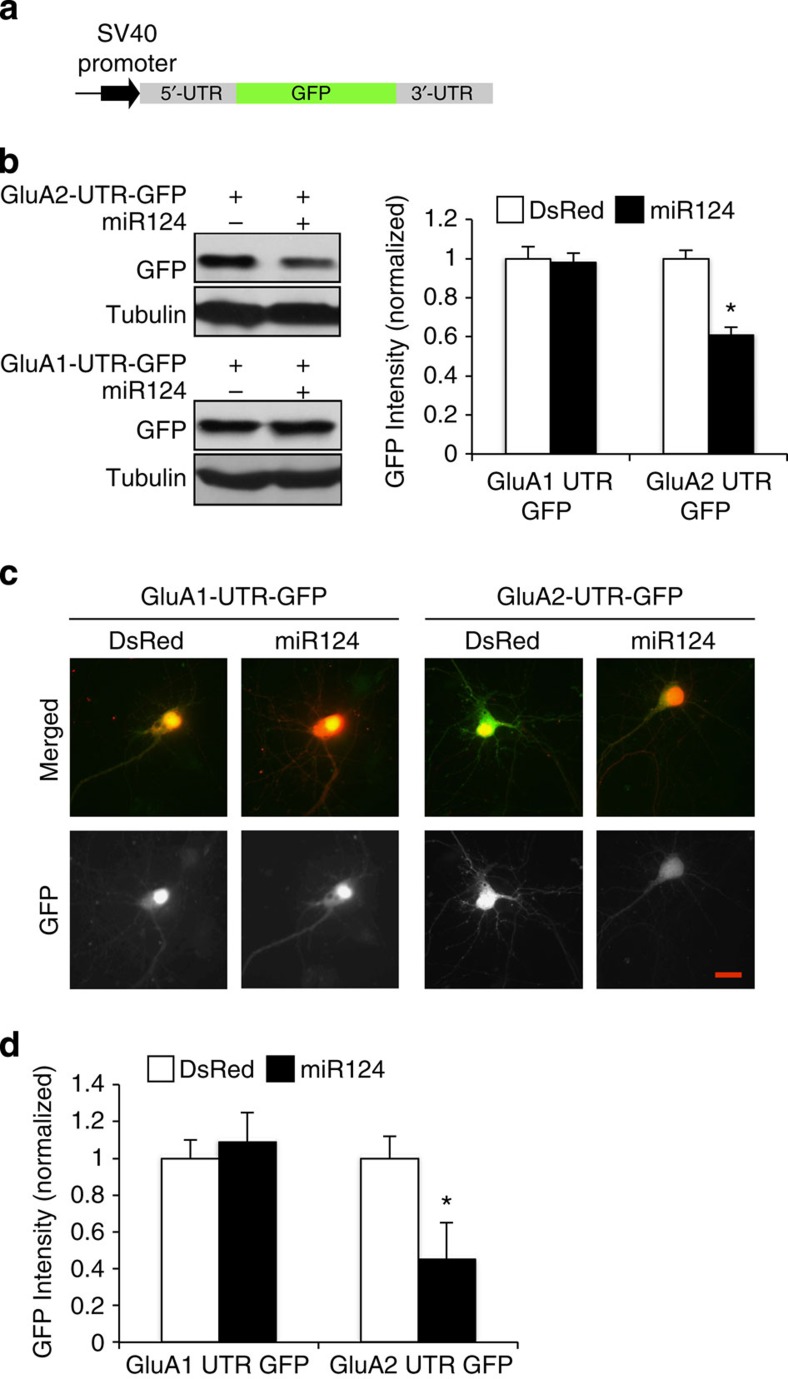
The UTR of GluA2 mRNA is required for miR124 effect on protein expression. (**a**) Construction of a GFP indicator. GFP sequence was flanked by 5′- and 3′-UTR from GluA2 or GluA1 mRNA. Alterations in GFP intensity indicate the effect of miR124 targeting the 3′-UTR. (**b**) HEK cells were transfected with miR124 and GFP-UTR. Western blotting showed a decrease of GluA2-UTR-GFP, but not GluA1-UTR-GFP. Tubulin was probed as control. Bar graphs represent mean±s.e. *n*=3 experiments, *P<0.05. (**c**,**d**) Hippocampal neurons were transfected with miR124, together with GFP-GluA1-UTR, GluA2-UTP-GFP or DsRed as a control, respectively. GFP intensity was measured 2 days after transfection. Expression of miR124 reduced expression of GFP-GluA2-UTR, but not GFP-GluA1-UTR. Bar graphs represent mean±s.e. GluA1-UTR-GFP: *n*=16 cells, GluA2-UTR-GFP: *n*=17 cells, *P<0.05, *t*-test. Image scale bar, 10 μm.

MicroRNAs suppress protein translation via complementary binding with a sequence in the 3′-UTR of a target mRNA. To determine whether the 3′-UTR is responsible for the effect of miR124, we used a GFP reporter plasmid in which the GFP mRNA is flanked by 5′- and 3′-UTR of GluA2 or GluA1 (Fig. 2a). Two days after transfection of the reporter GFP with or without miR124 into HEK293T cells, cell lysates were subjected to western blot analysis for GFP levels. We found that in cells expressing GluA2-UTR-GFP, co-expression of miR124 caused a significant reduction in GFP amounts (Fig. 2b, 61±4% of DsRed control, *P*<0.05, *n*=3; *t*-test). However, the level of GFP in GluA1-UTR-GFP was not affected by miR124 (Fig. 2b, 98±5% of DsRed control, *n*=3). Further supporting the miR124 specificity for GluA2, levels of tubulin remained unchanged in all conditions. Next, we co-transfected the reporter GFP with miR124 in hippocampal cultures, and the GFP intensity was examined 2 days after transfection. Consistent with the findings in HEK cells, miR124 overexpression significantly reduced GluA2-UTR-GFP, but not GluA1-UTR-GFP (Fig. 2c,d, GluA2-UTR: 45±20% of DsRed control, *P*<0.05, *n*=16; GluA1-UTR: 109±16% of DsRed control, *P*>0.05, *n*=17; *t*-test). Together, these results indicate that miR124 regulates GluA2 expression via an interaction with the GluA2 3′-UTR.

### Neutralization of miR124 increases GluA2 expression

**Figure 3:**
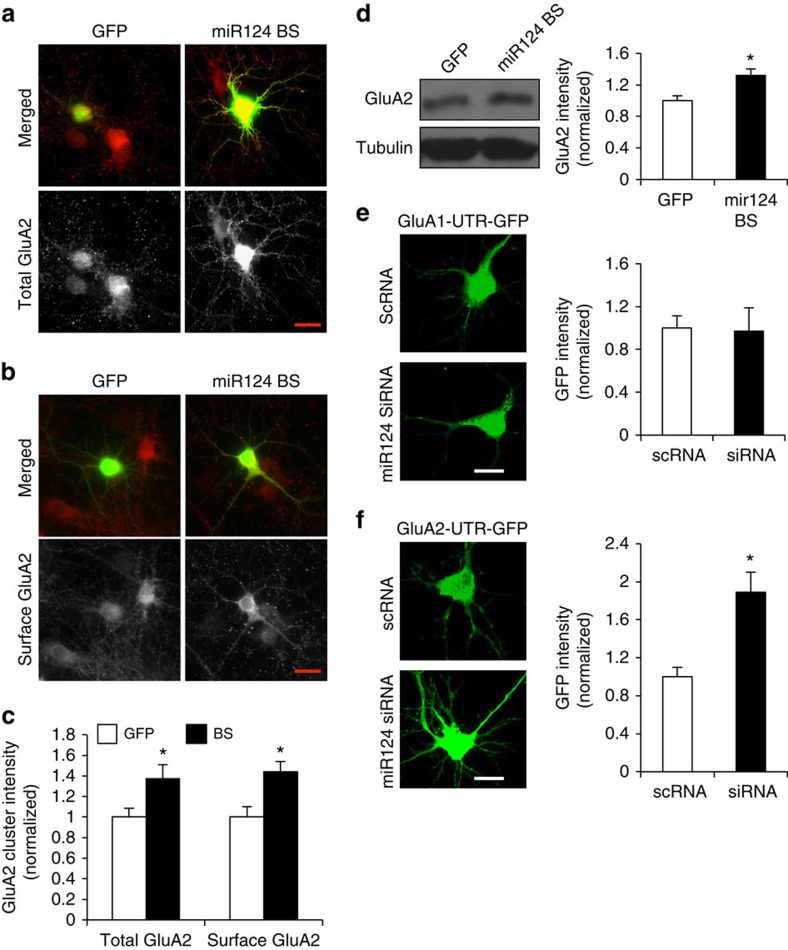
Sequestration of miR124 increases GluA2 expression. (**a**–**c**) Cultured hippocampal neurons were transfected with a miR124-neutralizing sponge BS (containing GFP) at DIV12 and GluA2 was immunostained 2 days later. GFP was transfected as a control. In cells expressing miR124 BS, both the total and cell surface GluA2 puncta intensity were increased. Bar graphs represent mean±s.e. Total GluA2: *n*=430 puncta from 16 cells; Surface GluA2: *n*=385 from 16 cells, *P<0.05, *t*-test. Image scale bars, 10 μm. (**d**) Cortical neurons were infected with BS lentivirus at DIV12 and cell lysates were analysed by western blots at DIV16 for GluA2 protein levels. miR124 BS-infected cells showed increased levels of GluA2. Tubulin was probed as control. Bar graphs represent mean±s.e., *n*=3, *P<0.05, *t*-test. (**e**,**f**) Hippocampal neurons were transfected with siRNA against miR124, together with GluA1-UTR-GFP or GluA2-UTR-GFP. Scrambled siRNA (scRNA) was used as a control. GFP intensity was measured 2 days after transfection. Inhibition of miR124 selectively increased expression of GluA2-UTR-GFP. Bar graphs represent mean±s.e., GluA2: *n*=21 cells; GluA1: *n*=22 cells, *P<0.05, *t*-test. Image scale bars, 10 μm.

Next, we examined the effect of miR124 suppression on GluA2 expression. To neutralize endogenous miR124, we transfected hippocampal neurons with a miR124-binding sponge (BS) construct. The sponge contains two tandem repeats of a sequence complementary to miR124, so that when overexpressed, it will dominantly bind to and sequester miR124 so as to avoid its interaction with *de novo* targets. Expression of miR124 BS markedly increased the puncta intensity of both total and the surface GluA2 puncta in hippocampal neurons (Fig. 3a–c, total: 137.2±13.5% of GFP control, *P*<0.05, *n*=430 puncta from 15 cells; surface: 144.2±9.7% of GFP control, *P*<0.05, *n*=385 puncta from 14 cells; *t*-test). This result suggests the existence of constitutive suppression of GluA2 expression by miR124 during basal conditions. To further confirm the effect of miR124 BS on the total cellular GluA2 amount, lysates of BS-infected neuron cultures were examined by western blot. Consistent with immunostaining data, we detected a significant increase in GluA2 protein levels in BS transfected neurons (Fig. 3d, 132±8% of GFP control, *P*<0.05, *n*=3; *t*-test). As a control, the BS miR124 did not alter the expression of total GluA1 levels (Supplementary Fig. 3, 104±5% of GFP control, *P*>0.05, *n*=10; *t*-test). We also transfected neurons with the UTR constructs together with siRNA against miR124. Similar to the effect of the BS, transfection of the miR124 siRNA led to an increase in GluA2-UTR-GFP expression (Fig. 3e,f, GluA2: 189±21% of scRNA control, *P*<0.05, *n*=21 neurons; GluA1: 97±22% of scRNA control, *P*>0.05, *n*=22 neurons; *t*-test). miR124 specificity was also examined by co-transfecting neurons with mir124 (containing DsRed), or a scrambled control (containing DsRed), with GluA2-UTR-GFP. In support of a role for GluA2-UTR in sequestering miR124, co-transfection of GluA2-UTR-GFP+miR124 was sufficient to block the miR124-mediated decrease in GluA2 (Supplementary Fig. 4, *n*=10 neurons each). To examine other possible synaptic targets for miR124, we examined the GABA α1 receptor subunit (α1R) and the NMDA receptor subunit GluN1 after suppression of miR124. Treatment with the miR124 inhibitor did not affect expression of α1R (Supplementary Fig. 5A and Supplementary Fig. 5B, 105±4% of scRNA control, *P*>0.05, *n*=10) or GluN1 (Supplementary Fig. 5C and Supplementary Fig. 5D, 96±3% of scRNA control, *P*>0.05, *n*=11; *t*-test), indicating the specificity for miR124 in the regulation of GluA2 expression.

### miR124 expression promotes the formation of GluA2-lacking AMPARs

**Figure 4:**
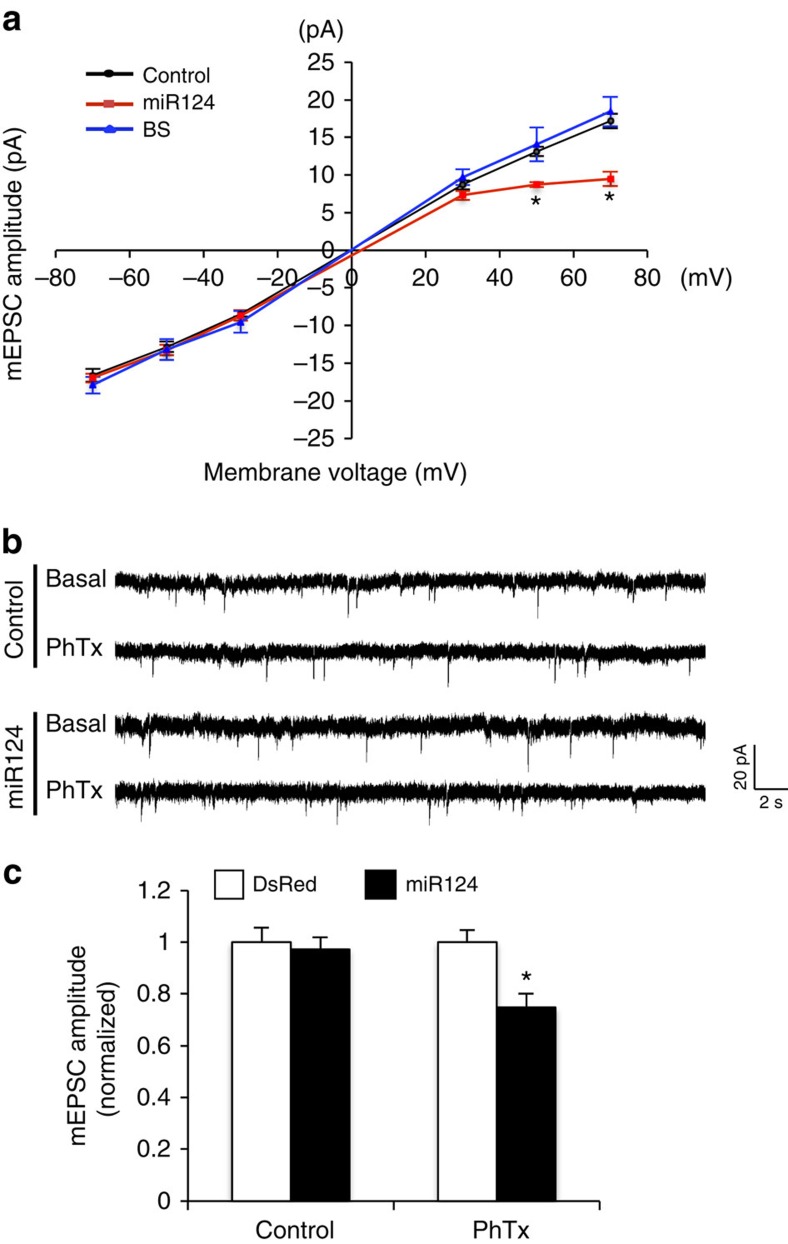
miR124 expression leads to biogenesis of GluA2-lacking AMPARs. (**a**) I–V curve of AMPAR-mediated mEPSCs. In hippocampal neurons transfected with miR124, BS or DsRed as a control, mEPSCs were recorded at indicated voltages. miR124-expressing neurons show an inwardly rectified I–V curve. *n*=5–7 cells. (**b**,**c**) miR124-expressing cells show higher sensitivity to PhTX. In DsRed control neurons, application of CP-AMPAR-specific antagonist PhTx had no effect on mEPSCs. In neurons transfected with miR124, PhTx significantly reduced mEPSC amplitude. Bar graphs represent mean±s.e., *n*=5–8 cells for each bar, *P<0.05, *t*-test.

To directly examine whether miR124 promotes the formation of GluA2-lacking CP-AMPARs, we measured the current–voltage relationship (I–V curve) of mEPSCs. Inward rectification of the I–V curve at positive membrane potentials is a signature feature of CP-AMPARs^40^. mEPSC analysis revealed a typical rectification of the I–V curve in cells transfected with miR124, whereas the control cells showed a linear I–V relationship (Fig. 4a). We also transfected neurons with the sponge construct BS to inhibit miR124 function. A normal linear I–V relationship was maintained in BS-expressing neurons (Fig. 4a), suggesting that most AMPARs are GluA2-containing during basal conditions. Furthermore, we examined the mEPSC sensitivity to PhTx, an antagonist specific for CP-AMPARs. In control neurons, application of PhTx had no effect on mEPSCs. In contrast, in neurons transfected with miR124, PhTx treatment caused a marked reduction in mEPSC amplitude (Fig. 4b,c). In addition, in miR124 transfected neurons, mEPSCs showed a faster decay time indicative of the presence of GluA2-lacking AMPARs (Supplementary Fig. 6A, 0.83±3% of control, *n*=6, *P*<0.05; *t*-test), while no change in mEPSC frequency was observed (Supplementary Fig. 6B, 0.91±20% of control, *n*=6, *P*>0.05; *t*-test). These results strongly indicate that miR124 expression results in the biogenesis of CP-AMPARs in neurons.

### miR124 is required for HSP

**Figure 5:**
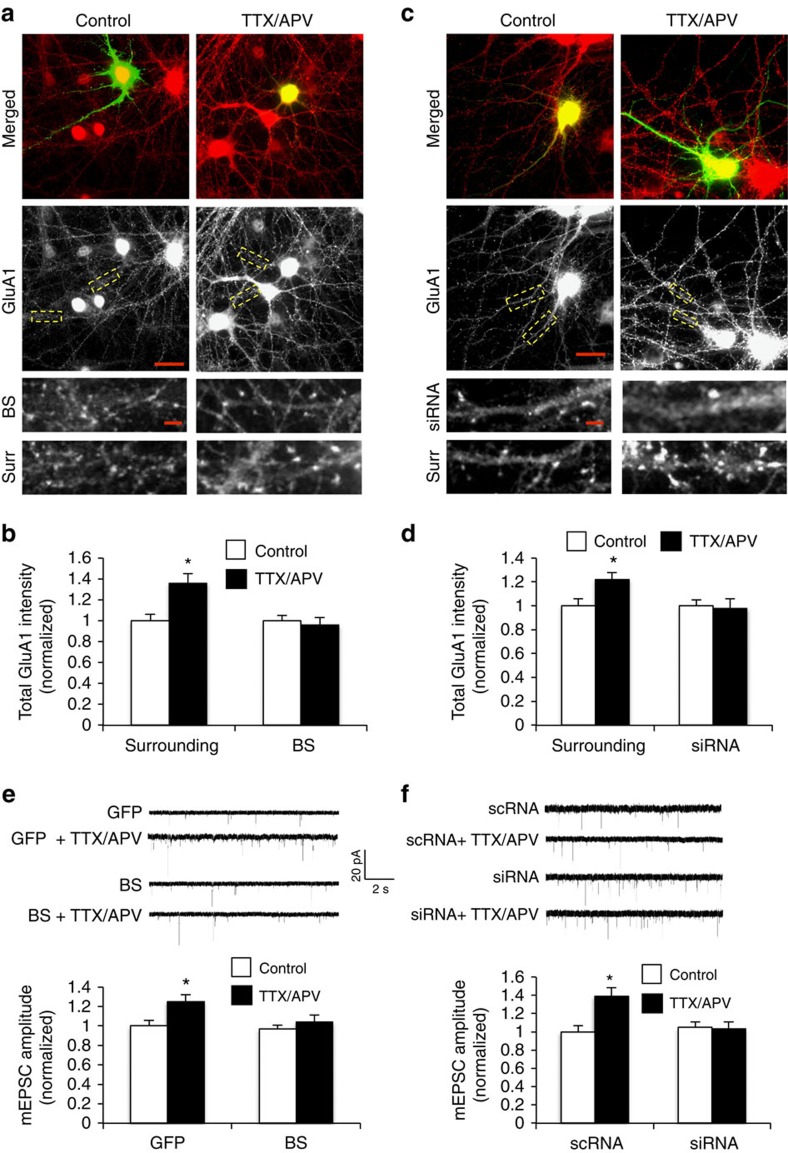
miR124 is required for the expression of inactivity-dependent homeostatic synaptic plasticity. (**a**,**b**) Hippocampal neurons were transfected with BS (with GFP) or GFP alone as control. One day after transfection, cells were incubated with TTX/APV for 15 h and immunostained for GluA1 (red). TTX/APV treatment increased GluA1 synaptic cluster intensity in non-transfected neighbouring surrounding cells (Surr), but not in neurons expressing miR124 BS. Segments of dendrites were enlarged for clarity (Fig. 6a, bottom). Bar graphs represent mean±s.e., surrounding cells: *n*=272 puncta from 15 cells, pBS transfected: 245 puncta from 15 cells, *P<0.05, *t*-test. Scale bars, 10 μm (full images), 3 μm (dendrites). (**c**,**d**) Neurons were transfected with miR124-specific siRNA for 1 day and then incubated with TTX/APV for 15 h. Inactivity induced an increase in GluA1 puncta intensity in the surrounding non-transfected neurons (Surr), but not cells expressing siRNA. Bar graphs represent mean±s.e., non-transfected surrounding: *n*=326 puncta from 16 cells, siRNA transfected: *n*=377 puncta from 16 cells, *P<0.05, *t*-test. Scale bars, 10 μm (full images), 3 μm (dendrites). (**e**) Hippocampal neurons were transfected with BS (containing GFP) or GFP as a control for 1 day, followed by TTX/APV incubation for 15 h before recordings. Amplitude of AMPAR-mediated mEPSCs was increased in the GFP control, but not BS-expressing cells. Bar graphs represent mean±s.e., *n*=7 cells, *P<0.05, *t*-test. T/A, TTX+APV. (**f**) mEPSC recordings in hippocampal neurons transfected with GFP together with miR124 siRNA, or with scrambled RNA as a control. siRNA abolished TTX/APV-induced homeostatic increase in mEPSC amplitude. Mean±s.e., *n*=10–14 cells, *P<0.05, *t*-test. T/A, TTX+APV.

It has been previously shown that CP-AMPARs are expressed during neuronal inactivity, and blockade of CP-AMPARs leads to the abolishment of homeostatic regulation. We therefore hypothesized that miR124, via downregulating GluA2 expression, plays an important role in the initiation of the homeostatic response. If so, the expression of miR124 should be coupled with neuronal activity. To test this idea, we performed RT–PCR to examine the effect of neuronal inactivity on miR124 abundance. We found that incubation of hippocampal neurons with TTX/APV for 15 h resulted in 1.7-fold increase in the amount of miR124 (Supplementary Fig. 7), suggesting a role for miR124 in HSP under neuronal activity deprivation. To test this possibility, we investigated whether suppression of miR124 blocks HSP. DIV 11 cultured hippocampal neurons were transfected with the specific BS to neutralize endogenous miR124. One day after transfection, cells were then incubated with TTX/APV for 15 h. Given that HSP is expressed by an elevated expression of GluA1-containing AMPARs, we immunostained GluA1 following TTX/APV incubation. The same batch of cultured neurons was transfected with an empty vector as control. As expected, TTX/APV reliably induced a homeostatic increase in GluA1 synaptic accumulation. However, when endogenous miR124 was blocked by BS, TTX/APV failed to trigger homeostatic changes in AMPAR expression (Fig. 5a,b, non-transfected neighbouring cells: 136±9% of non-treatment control, *P*<0.05, *n*=272 puncta from 15 cells; BS transfected neurons, 96±7% of non-treated control, *P*>0.05, *n*=245 puncta from 15 cells; *t*-test). To further confirm the requirement of miR124 function, we also transfected neurons with an inhibitor siRNA against miR124. Similar to the effect of the BS, transfection of the miR124 inhibitor led to a complete abolishment of TTX/APV-induced homeostatic elevation in GluA1 puncta intensity (Fig. 5c,d, non-transfected neighbouring cells: 122±6% of the control, *P*<0.05, *n*=326 puncta from 12 cells; BS transfected, 98±8% of the control, *P*>0.05, *n*=377 puncta from 10 cells; *t*-test). Similar to GluA1, 15 h TTX/APV also induced a significant increase in synaptic GluA2 both in the control and miR124-transfected neurons (Supplementary Fig. 8A, surrounding: 137.7%±8.2%, *n*=379 puncta from 17 cells; miR124: 133.4%±5.7%, *n*=425 puncta from 14 cells; *t*-test). In addition, the homeostatic change in GluA2 was abolished by BS (Supplementary Fig. 8B, surrounding: 135.9%±8.3%, *n*=483 puncta from 16 cells; BS: 94.8%±13.5%, *n*=527 puncta from 12 cells; *t*-test). These immunostaining data indicate the requirement of miR124 function in homeostatic AMPAR expression.

To further investigate the role of miR124 in homeostatic regulation of synaptic transmission, AMPAR-mediated mEPSCs were recorded from hippocampal cultures transfected with BS or miR124 siRNA as above. Consistent with previous studies, a significant increase in mEPSC amplitude was induced following 15 h incubation with TTX/APV. In contrast, no significant alterations in mEPSC amplitude by TTX/APV treatment was detected in cells expressing either the BS construct or the siRNA inhibitor of miR124 (Fig. 5e,f, BS: 104±7%, *n*=6, *P*>0.05; siRNA: 103±8%, *n*=6, *P*>0.05; *t*-test). Together, these findings indicate that miR124 function is necessary for the expression of HSP.

To determine the role of CP-AMPARs for HSP, neurons were transfected with miR124 and incubated with a mixture of TTX/APV+PhTx for 12 h. We found that HSP was completely abolished in both the control and miR124-expressing neurons (Supplementary Fig. 9 and Supplementary Table 1, control TTX/APV/PhTX=0.95±0.05 of control, *n*=6, *P*>0.05; miR124 TTX/APV/PhTx=0.97±0.05 of control, *n*=7, *P*>0.05; *t*-test), indicating that GluA2-lacking AMPARs are required for HSP expression.

### miR124 facilitates the initiation of HSP

**Figure 6:**
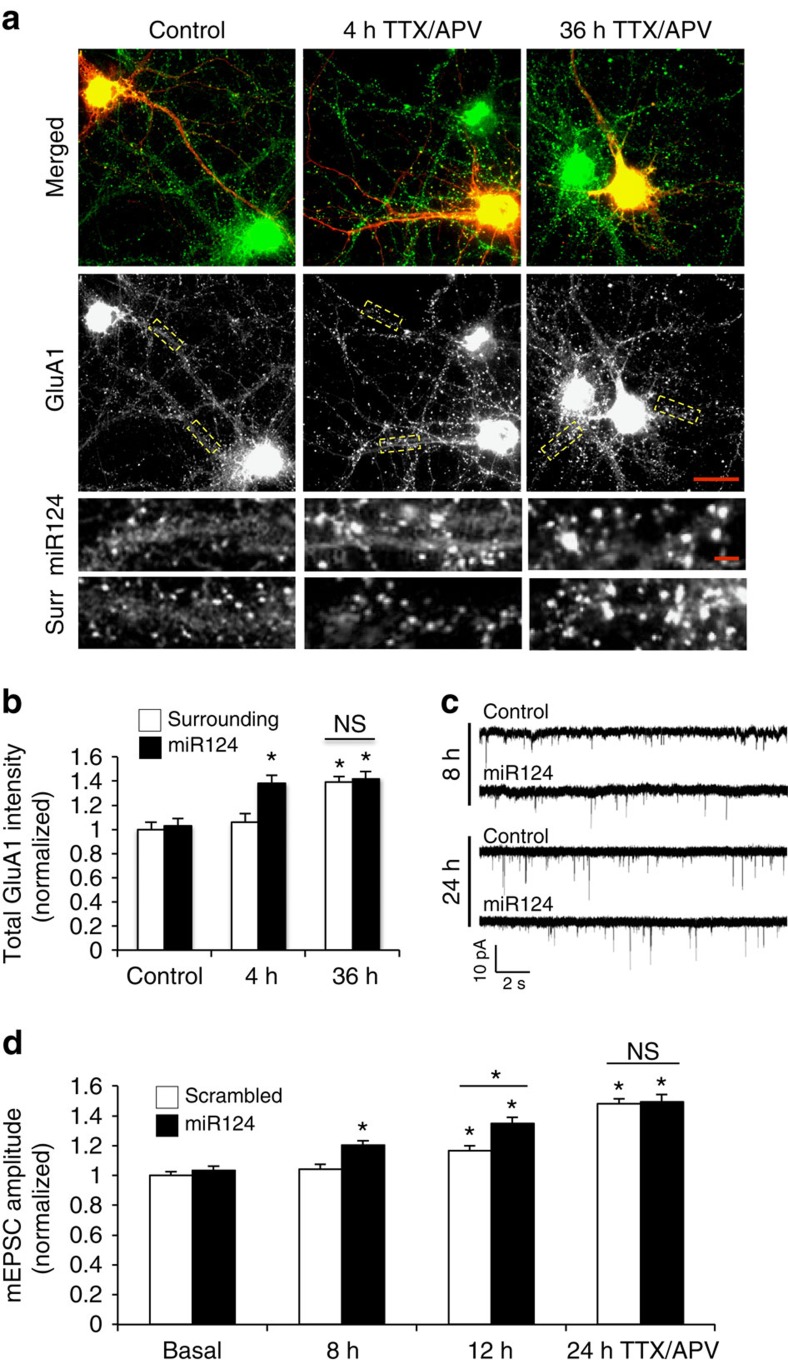
miR124 facilitates expression of homeostatic synaptic plasticity. (**a**,**b**) Hippocampal neurons were transfected with miR124 (red) for 2 days, followed by incubation with TTX/APV for 4 or 36 h. Immunostaining of GluA1 (green) revealed an increase in GluA1 puncta intensity at 4 h only in miR124-transfected, but not surrounding (Surr) cells. At 36 h treatment, both miR124-transfected and surrounding neurons showed an increase in GluA1 intensity. Bar graphs represent mean±s.e. Non-treated control, *n*=437 puncta from 16 cells; 4 h treatment, *n*=461 puncta from 16 cells; 36 h treatment, *n*=378 puncta from 16 cells, *P<0.05, *t*-test. Scale bars, 10 μm (full images), 3 μm (dendrites). (**c**,**d**) mEPSC recordings from neurons transfected with miR124 (containing DsRed) or DsRed alone as control. After 8 h of incubation with TTX/APV, mEPSC amplitude was not changed in control neurons, but significantly increased in neurons overexpressing miR124. At 12 and 24 h treatment, both groups showed homeostatic increase in mEPSC, but miR124 cells had stronger response than the time control at 12 h. Representative mEPSC traces were shown in **c**. Bar graphs (**d**) represent mean±s.e., *n*=5–6, *P<0.05, *t*-test.

If miR124 leads to the formation of CP-AMPARs, which are required for the expression of homeostatic plasticity, we reasoned that the homeostatic response might be facilitated by miR124. We therefore explored the role of miR124 in the time course of the induction of the homeostatic response. In most studies, significant homeostatic responses were detected following 12–48 h incubation with TTX or TTX/APV. We reasoned that if GluA2-lacking receptors serve as a rate-limiting factor that allows inactivity to initiate homeostatic events, increased amount of miR124 should be able to expedite homeostatic induction via CP-AMPARs. To test this idea, 48 h after miR124 transfection, hippocampal neurons were incubated with TTX/APV for various periods of time. With an incubation time of 4 h, a significant increase in GluA1 puncta intensity was detectable in neurons transfected with miR124 (Fig. 6a,b, 4 h TTX/APV miR124 neurons: 138±7% of control, *P*<0.05, *n*=461 puncta from 16 cells; *t*-test). In contrast, for non-transfected neighbouring cells in the same culture, no homeostatic increase in GluA1 was detected (Fig. 6a,b, non-treated miR124 neurons: 103±6% of non-transfected cells, *P*>0.05, *n*=437 puncta from 18 cells; 4 h TTX/APV surrounding neurons: 106±7% of control, *P*>0.05, *n*=417 puncta from 15 cells; *t*-test). However, when GluA1 accumulation was examined after 36 h TTX/APV treatment, the same level of increase was observed in both miR124 transfected and non-transfected cells (Fig. 6a,b, 36 h miR124 neurons: 102±4% of non-transfected surrounding control, *P*>0.05, *n*=378 puncta from 10 cells; *t*-test). These data indicate that pre-expression of miR124 facilitates the expression of homeostatic plasticity, but not the strength or magnitude of the response. With a sufficient period of activity suppression, the homeostatic response will reach the same extent between miR124-transfected and the control neurons.

Using a similar time course of TTX/APV treatment, we also measured mEPSCs as an indicator of the functional homeostatic response. In our cultures, homeostatic increases in mEPSC amplitude could be reliably obtained after 12 h TTX/APV incubation, but no significant increase was detected when we shortened the recording time to 8 h. However, in neurons transfected with miR124, a significant increase in mEPSC amplitude was induced 8 h after activity inhibition (Fig. 6c,d, DsRed control: 104±4% of basal control, *n*=6; miR124: 117±3%, *n*=6, *P*<0.05; *t*-test). A higher response was detected in miR124 neurons at 12 h treatment (DsRed control: 116±3% of basal control, *n*=6; miR124: 131±3%, *n*=6, *P*<0.05; *t*-test). At 24 h TTX/APV incubation, we detected a more robust, but comparable level of increase in mEPSC amplitude in both the control and miR124-transfected neurons (Fig. 6c,d, DsRed: 143±4% of basal control, *n*=5; miR124: 148±4%, *n*=5; *t*-test), indicating a time-dependent saturation. On mEPSCs from neurons of 12 h TTX/APV treatment, analysis of the cumulative probability of amplitude and frequency showed typical homeostatic scaling of mEPSC amplitude with no change in inter-event intervals (Supplementary Fig. 6C,D).

To determine the contribution of normal versus CP-AMPARs in HSP, we added PhTx at the time of recording following 12 h TTX/APV incubation in cells transfected with miR124, or a scrambled control. We found that after induction of HSP, application of PhTx during recordings led to a significant decrease in mEPSC amplitude in neurons overexpressing miR124, but not in the control cells. However, in the presence of PhTx, the remaining currents were still significantly higher than the currents under basal conditions. These results indicate that HSP is expressed by GluA2-containing AMPARs under normal conditions, whereas both GluA2-containing and GluA2-lacking AMPARs contribute to HSP under the condition of miR124 overexpression (Supplementary Fig. 10 and Supplementary Table 2, 12 h TTX/APV control=1.22±0.05 of basal control, *n*=6, *P*<0.01; 12 h TTX/APV miR124=1.38±0.05, *n*=6, *P*<0.001; 12 h TTX/APV control+PhTx=1.14±0.04, *n*=9, *P*<0.05; 12 h TTX/APV miR124+PhTx=1.19±0.04, *n*=9, *P*<0.01; *t*-test).

### The transcription factor EVI1 regulates miR124 expression

**Figure 7:**
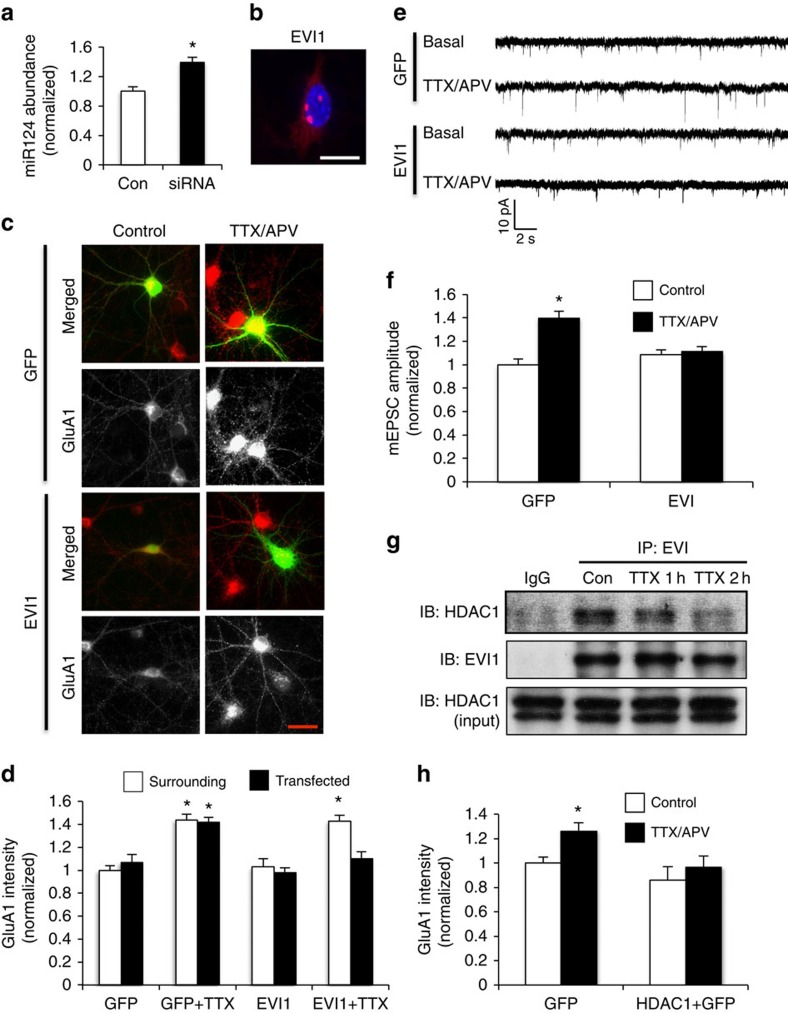
The transcription factor EVI1 regulates miR124 expression and homeostatic plasticity. (**a**) EVI1-specific siRNA was transfected in HEK cells for 2 days, and miR124 expression levels were quantified by RT–PCR. Bar graphs represent mean±s.e., *n*=3, *P<0.05, *t*-test. (**b**) Hippocampal neurons were immunostained for EVI1 (red), and the nuclei were labelled with Hoechst (blue). Scale bar, 10 μm. (**c**,**d**) Hippocampal neurons were transfected with EVI1 plus GFP, or GFP alone, and were incubated with TTX/APV for 15 h. Immunostaining of GluA1 (red) showed an increase in GluA1 puncta intensity in control, but not EVI1-transfected neurons. Bar graphs represent mean±s.e. GFP-only: *n*=694 puncta from 20 cells; neighbouring: *n*=547 puncta from 20 cells; EVI1: *n*=531 puncta from 20 cells; neighbouring: *n*=482 puncta from 18 cells, *P<0.05, *t*-test. T/A, TTX+APV. (**e**,**f**) mEPSC recordings in neurons transfected with GFP only, or together with EVI1. The 15 h TTX/APV treatment induced homeostatic increase in mEPSC amplitude in neurons expressing GFP, but not in cells transfected with EVI1. Image scale bar, 10 μm. (**g**) Co-immunoprecipitation of EVI1 and HDAC1. Using lysates from cortical neurons incubated with TTX/APV for 1 and 2 h, EVI1 complexes were isolated by immunoprecipitation and probed for HDAC1. EVI1 co-immunoprecipitated with HDAC1, and the association was negatively regulated by neuronal inactivity. T/A, TTX+APV. (**h**) HDAC1 expression blocks homeostatic response. In neurons transfected with GFP or together with HDAC1, 15 h incubation with TTX/APV induced homeostatic increase in GluA1 synaptic expression in GFP control cells, but not neurons overexpressing HDAC1. *n*=600–800 synapses from 15 cells, *P<0.05, *t*-test.

Our data indicate a crucial role for miR124 in HSP. We wanted to further understand how miR124 expression is regulated in an activity-dependent manner. In non-neuronal cells, the transcription factor EVI1 has been shown to regulate miR124 expression^41^. EVI1 binds to the regulatory region of the miR124 gene to suppress its transcription^42^,^43^. Consistent with this, in HEK cells, we found that knockdown of EVI1 by an EVI1-specific siRNA led to increased miR124 expression (Fig. 7a). We hypothesized that activity deprivation may lead to downregulation of EVI1-dependent suppression, resulting in miR124 expression and the biogenesis of CP-AMPARs. To test this idea, we first examined EVI1 subcellular localization. Immunostaining in hippocampal neurons showed that EVI1 was mainly localized at the soma with further enrichment in the nucleus in a punctate pattern (Fig. 7b). Next, we examined the interaction between EVI1 and the miR124 promoter. Using cortical neuron lysates, we immunoprecipitated EVI1 and performed ChIP assays to examine the presence of the miR124 promoter sequence by PCR amplification. We found that the miR124 promoter was indeed co-immunoprecipitated with EVI1 (Supplementary Fig. 11), confirming an association of EVI1 with the regulatory region in the miR124 gene in neurons.

### Involvement of EVI1 and HDAC1 in HSP

Having established that EVI1 regulates miR124 expression, we investigated the role of EVI1 in HSP. In neurons co-transfected with EVI1 and GFP, or GFP alone as a control, 15 h incubation with TTX/APV caused a significant increase in GluA1 puncta in both the GFP-only control and neighbouring, non-transfected cells (Fig. 7c,d, GFP-only: 142±4% of non-treated control, *P*<0.05, *n*=694 puncta from 18 cells; neighbouring cells: 144±5% of non-treated control, *P*<0.05, *n*=547 puncta from 17 cells; *t*-test). In contrast, the same treatment failed to induce a significant change in GluA1 in EVI1-transfected cells (EVI1: 110±6% of non-treated control, *P*>0.05, *n*=531 puncta from 16 cells; neighbouring cells: 143±5% of non-treated control; *t*-test). Consistent with this, in EVI1 transfected neurons, electrophysiological recordings of mEPSCs revealed a typical homeostatic increase in mEPSC amplitude in non-transfected cells, but not in cells overexpressing EVI1 (Fig. 7e,f).

EVI1 has been suggested to associate with HDAC1, a histone deacetylase that has an important role in transcriptional suppression via histone deacetylation^44^,^45^. We reasoned that the EVI1–HDAC1 interaction, and thus the inhibitory effect in gene transcription may be controlled by neuronal activity. We therefore immunoprecipitated EVI1 from lysates of cultured cortical neurons, and probed for HDAC1. Indeed, HDAC1 was successfully co-immunoprecipitated with EVI1. Importantly, activity deprivation by TTX/APV treatment induced a reduction in the EVI1–HDAC1 interaction (Fig. 7g). The interaction between EVI1 and HDAC1 was also confirmed by reversed co-immunoprecipitation assays (Supplementary Fig. 12). To further determine the role of HDAC1 in homeostatic regulation, hippocampal neurons were transfected with HDAC1, then incubated with TTX/APV for 15 h. We found that in contrast to the homeostatic increase in synaptic GluA1 in GFP-transfected control cells, inactivity treatment failed to cause changes in neurons overexpressing HDAC1 (Fig. 7h). Taken together, these findings are in agreement with an important role for EVI1 and HDAC1 in activity deprivation-induced HSP, presumably via regulation of miR124 expression and the generation of CP-AMPARs.

## Discussion

HSP is crucial for the maintenance of neuronal and neural circuit stability, but the underlying cellular and molecular mechanisms remain less well understood. Our data identify miR124 as an important mediator in the expression of the homeostatic response. miR124 specifically suppresses GluA2 expression via targeting on the 3′-UTR of GluA2 mRNA, leading to the biogenesis of CP-AMPARs. During our paper preparation and revision for publication, a few studies have been published showing the role of miR124 on GluA2 expression^46^,^47^, consistent and in support of our findings. We find that blockade of miR124 function abolishes the inactivity-induced homeostatic response in AMPAR synaptic accumulation and mEPSC amplitude, strongly indicating a key role for miR124 in HSP. Immunostaining of GluA1, which was used to indicate AMPAR levels, showed a significant increase during HSP. Thus, the increased AMPARs are GluA1-containing. Following HSP induction, addition of PhTx had little effect on the elevated AMPAR-mediated currents, indicating that most of the AMPARs are also GluA2-containing. Therefore, normal AMPARs containing both GluA1 and GluA2 are inserted at synapses during HSP expression. In comparison, in neurons overexpressing miR124, PhTx resulted in a marked decrease in AMPA currents after neuronal inhibition, suggesting that both normal and GluA2-lacking AMPARs contribute to HSP with the overexpression of miR124. Likely, under physiological conditions, neuronal inactivity triggers transient upregulation in miR124, leading to temporary formation of GluA2-lacking AMPARs for HSP initiation, followed by enhanced synaptic insertion of normal, GluA1/GluA2-containing AMPARs for HSP maintenance^21^. During HSP expression, the switch from CP-AMPARs to normal AMPARs has been observed in many studies^17^,^21^,^48^. For instance, during TTX or APV-induced HSP, mEPSC currents are highly sensitive to the CP-AMPAR-specific antagonist Naspm. However, 24 h after activity inhibition, Naspm only has a minimal effect on mEPSC amplitude^17^, indicating that both GluA2-lacking and GluA2-containing AMPARs are utilized for the homeostatic upregulation of synaptic strength^48^. Indeed, knockdown of GluA2 abolishes homeostatic response^49^. Consistent with the involvement of GluA2-containing AMPARs in HSP expression, the amounts of both GluA1 and GluA2 are increased in neuronal inactivity-dependent scaling^8^,^50^. However, in retinoic acid-induced HSP, the homeostatic increase in mEPSC amplitude seems to result solely from CP-AMPARs because the HSP response can be completely abolished by PhTx^18^. Thus, the relative contribution of CP-AMPARs in HSP varies depending on the paradigms, and probably also on the time course of HSP expression and/or the developmental stage of the neurons^51^.

Interestingly, although miR124 facilitates the expression of homeostatic plasticity, miR124 overexpression alone does not induce the response. This finding indicates that miR124, and thus CP-AMPARs, are necessary but not sufficient for the initiation of the homeostatic response. We hypothesize that additional factor(s) induced by activity suppression, such as retinoic acid^18^, are required. It is also possible that miR124 initiates some HSP-antagonizing processes, which can be suppressed by TTX/APV. Similar to the complex signaling cascades in Hebbian synaptic plasticity, future studies are expected to identify additional key molecules involved in HSP.

miR124 is one of the most abundant microRNAs in the nervous system. In the rat brain, miR124 levels increase 13-fold from E12 to E21 (ref. 30), indicating a role in neural development. Indeed, miR124 has been shown to be critically involved in neurogenesis, migration, morphogenesis and neuronal cell death^38^. An appropriate level of miR124 seems important for brain function^52^. In mice, knockout of the cAMP sensor protein EPAC results in abnormal synaptic plasticity, deficits in spatial learning and social interactions, accompanied by increased expression of miR124. Remarkably, suppression of miR124 completely rescues, whereas miR124 expression mimics, these phenotypes^53^. This is consistent with the key role of miR124 in long-term plasticity of synaptic function^39^. miRNAs are implicated in neurodevelopmental disorders such as Fragile X syndrome and neurodegenerative diseases including Alzheimer's disease^25^,^54^,^55^,^56^. Alterations in miR124 show sensitivity to brain injuries and can be a biomarker for stroke^57^,^58^. Consistent with this, a recent study shows that expression of GluA2/3/4-containing AMPARs is regulated by miR124, which is implicated in the pathology of frontotemporal dementia^46^.

Glutamate receptors and their downstream signaling proteins are controlled by microRNAs. MiR219 has been shown to regulate NMDA receptor-dependent behavior via targeting on calcium/calmodulin-dependent protein kinase II (CaMKII)^59^. In Drosophila neuromuscular junctions, where the glutamatergic system is used for transmission, suppression of miRNA production by postsynaptic knockdown of dicer-1 results in elevated amounts of AMPA receptor subunits^60^. In demyelinated hippocampal neurons, an increase in miR124 is correlated with a reduction in GluA1 and GluA2 (ref. 61).

Our data demonstrate that miR124 expression is coupled to neuronal activity. Suppression of neuronal activity by TTX/APV induces an increase in the amount of miR124, supporting a role for miR124 in the homeostatic response. To further understand the molecular regulatory process, we find that in neurons, miR124 transcription is controlled by an inhibitory transcription factor EVI1. In peripheral cells and cancer cells, EVI1 has been shown to bind to the promoter region of miR124 and suppresses its transcription. In neurons, EVI1 is highly expressed in the nucleus forming a few punctate hot spots, presumably sites of specific promoter regions, including that for miR124. EVI1 is also distributed in the cytosol and the dendrites at a reduced level. In neurons, the exact molecular events by which EVI1 suppresses miR124 expression have not been studied. Earlier studies in non-neuronal cells suggest that EVI1 can bind to the transcription suppressor CtBP, which can then bind to the deacetylase HDAC1. Histone acetylation plays a key role in the epigenetic regulation of gene transcription, in which acetylation of histones activates gene transcription. The reverse process, histone deacetylation by HDACs, is a well-known mechanism for gene silencing. In support of this molecular machinery, we find that in neurons EVI1 is indeed associated with HDAC1, and the interaction is weakened by neuronal activity suppression. How neuronal activity controls the EVI1–HDAC1 association remains unclear, but it could result from a change in protein association affinity via modifications such as protein phosphorylation. The role of EVI1 in neurons is probably to recruit HDAC1 to the proximity of a target gene, for example, miR124, via their association, so as to regulate the chromatin acetylation status. Importantly, overexpression of EVI1 or HDAC1 completely abolished inactivity-induced HSP, consistent with their inhibitory effects on miR124 expression.

## Methods

### Culture of primary hippocampal neurons

Hippocampi from E18 rat embryos (Sprague Dawley) were digested with papain (0.1 mg ml^−1^ in HBSS, 37 °C for 20 min), washed and triturated with a serological pipette. To ensure high-quality cell adhesion and growth, coverslips were pre-incubated in nitric acid overnight and thoroughly washed with four changes of large amounts of water every 2 h. After a 70% ethanol wash and flame sterilization, glass coverslips were coated with poly-L-lysine (Sigma, 0.1 mg ml^−1^) overnight and washed again before being placed into culture dishes containing plating medium. Neurons were counted and plated onto 60 mm Petri dishes containing five coverslips with 18 mm diameter and 0.1 mm thickness (0.7 × 10^6^ cells per 60 mm dish). The plating medium was MEM containing 10% fetal bovine serum (FBS), 5% horse serum (HS), 31 mg cysteine, 5 mM Glutamax and 1% P/S. 24 h after plating, the culture medium was replaced with feeding medium (neurobasal medium supplemented with 1% HS, 2% B-27, 2 mM Glutamax and 1% P/S). Thereafter, hippocampal neurons were fed twice a week with 2 ml feeding medium per dish until use. All the procedures involving animal use in this study were in compliance with the policies of the Institutional Animal Care and Use Committee (IACUC) at Boston University, following the National Institutes of Health (NIH) guide for the care and use of Laboratory animals (NIH Publications No. 8023, revised 1978). Approved experimental protocol number: 11–039. The number of animals used in this study has been minimized.

### Neuron and HEK cell transfection

Coverslips of 12-day-old hippocampal neurons were first transferred to a 12-well plate and transfected with Lipofectamine 2000 (Invitrogen) according to the manufacturer's protocol. For one coverslip, 0.8 μg of DNA and Lipofectamine (0.8 μl) were separately diluted with EBSS, combined and incubated at room temperature for 20 min. The DNA complex was then added to a well containing 0.5 ml of feeding medium and kept in the incubator. After 4 h incubation, the transfection medium was removed and replaced with fresh feeding medium until the next medium change or use for experiments.

HEK cells (ATCC) were cultured and split into six-well plates (1 million per well) to grow overnight before transfection. The transfection process for HEK cells was identical to that described for neurons except that 4 μl Lipofectamine 2000 was mixed with 4 μg target plasmid to transfect each well of cells. Medium was changed 4 h post transfection and HEK cells were further cultured an additional 24 h to ensure target protein expression before cells were collected for western blot analysis. HEK cells were cultured in the following medium: 1 × DMEM with 10% FBS, 1% P/S and 1% l-Glutamine. MiR124 and RFP were cloned into the pFUGW vector (Addgene, #14883) between the BamHI and EcoRI sites. GFP-GluA1 and GFP-GluA2 constructs were generous gifts from the Lu Chen Lab. The mouse GluA1 3′-UTR and GluA2 3′-UTR (Accession #s: NM-008165 and NM_013540) were amplified from mouse hippocampal cDNA. The UTR fragments and GFP were then inserted into pCI-Neo (Promega). The EVI1 construct was a generous gift from the Zhijian Qian lab. Two tandem repeats of the miR124 BS sequence (5′- TGGCATTCACAAGTGCCTTAA -3′) were cloned into pEGFP-N1. A pool of 4 miR124 siRNAs was purchased from Qiagen (Cat. #: GS406907).

### Total RNA extraction and quantitative real-time PCR

Total RNA was purified using TRIzol (Invitrogen) and treated with DNAse to remove DNA contamination from hippocampal cultures. Six micrograms total RNA is recovered from 60 mm culture dish based on the optical density at 260 nm. The optical density A260/A280 ratio is >1.9. To analyse the expression levels of the mature miR124 sequence (5′- UGUGUUCACAGUGGACCUUGAU -3′), 2 μg of total RNA was reverse transcribed and quantitative real-time PCR (qRT-PCR) was performed with a 7300 real-time PCR system (Applied Biosystems) using a TaqMan mir124 assay kit (Applied Biosystems. Cat. #: 4426961). Transcript levels were normalized to the mature U6 snRNA sequence (5′- GTGCTCGCTTCGGCAGCACATATACTAAAATTGGAACGATACAGAGAAGATTAGCATGGCCCCTGCGAGGATGACACGCAAATTCGTGAAGCGTTCCATATT -3′) using a micro RNA Taqman assay (Applied Biosystems, Cat. # 4427975).

### Immunocytochemistry

Hippocampal neurons were washed with ACSF and fixed with 4% paraformaldehyde/4% sucrose for 10 min on ice, permeabilized with 0.25% Triton X-100 (on ice, 10 min) or stained without permeabilization for surface labelling. Coverslips with neurons were blocked with 10% normal goat serum (NGS) in phosphate-buffered saline (PBS) for 1 h and then incubated with primary antibodies dissolved in 5% NGS in PBS for 2 h at room temperature. The cells were then washed four times with PBS and incubated with fluorescent Alexa Fluor-conjugated secondary antibodies (1:600, Thermo Fisher) for 1 h for visualization. For surface staining, live neurons were incubated with antibodies against the extracellular amino (N) termini of GluA2 (1:100) in culture medium in the incubator for 10 min. Plates were then placed on ice and washed four times with ACSF. After fixation, the cells were blocked and incubated with fluorescent secondary antibodies as above. The specificity of surface labelling was confirmed by a lack of intrasoma immunointensity and a lack of staining by incubation with GluA2 carboxy (C)-terminal antibodies.

The following primary antibodies were used: GluA1C (Rabbit, 1:400; homemade) and GluA1N (Mouse, 1:100; Millipore), EV1 (Rabbit, 1:400, Abcam); PSD-95 (Mouse, 1:400; Thermo Fisher); GluN1C (Mouse, 1:200; Millipore); GABA α1 (Rabbit, 1:600, Abcam).

### Co-immunoprecipitation and western blot

Two-week-old cultured cortical neurons were incubated with TTX/APV for 1–2 h and harvested in ice-cold lysis buffer (PBS supplemented with 1% Triton X-100, 0.5% deoxycholate, 0.1% SDS and 1:300 protease inhibitor cocktail containing AEBSF, Aprotinin, Bedysyin, E-64, Leupeptin and Pepstatin A, Sigma) and rotated at 4 °C for 1 h. Following centrifugation of the lysates at 14,000*g* for 15 min, supernatants were incubated overnight on rotation at 4 °C with anti-EVI1 antibodies, (1 μg, Abcam) followed by the addition of 40 μl of 50% slurry of protein A-Sepharose beads (Santa Cruz Biotechnology). Immunoprecipitates were washed three times with lysis buffer and resuspended in 30 μl of 2 × Laemmli buffer and denatured on a 95 °C heat block for 10 min. Immunoprecipitates were analysed by western blotting. The full western blots are shown as Supplementary Figs 13–16.

The following antibodies were used for western blot: GFP (Mouse, 1:500, Abcam); GluA1C (Rabbit, 1:1,000; homemade) and GluA1N (Mouse, 1:1,000; Millipore), EV1 (Rabbit, 1:1,000, Abcam); PSD-95 (Mouse, 1:1,000; Thermo Fisher); HDAC1 (Rabbit, 1:1,000, Cell Signaling).

### DNA chip assay

DIV14 cultured cortical neurons (2 × 10^7^) were washed once with 1 × PBS. Cell fixation was performed by the addition of 37% formaldehyde to 0.75% final concentration and rocking gently for 10 min at room temperature. One millilitre of 1.25 M glycine for every 9 ml crosslinking solution was added to quench the reaction. The cells were scraped into 5 ml cold PBS and washed with cold PBS and lysed with FA lysis buffer (50 mM HEPES-KOH pH 7.5, 140 mM NaCl, 1 mM EDTA pH 8, 1% Triton X-100, 0.1% sodium deoxycholate, 0.1% SDS, protease inhibitors) and sonicated 10 times with 20 s on/off. The sonicated lysate was then diluted 1:10 with RIPA buffer, and incubated with 2 μg EVI1 antibodies or negative control IgG for 1 h, followed by the addition of 30 μl protein A/G agarose beads for overnight on rotation. Antibody-bound EVI1 complexes were precipitated by centrifugation for 1 min at 2,000 g at 4 °C. Precipitates were washed four times with washing buffer. Antibody–protein complexes were eluted with freshly prepared, pre-heated elution buffer (1% SDS, 100 mM NaHCO3) at 65 °C. Sodium chloride was added to the elutions and input samples to a final concentration of 200 mM NaCl and heated at 65 °C for 4 h. RNase A and proteinase K were added to digest RNA and protein. A 2 μl sample was used for PCR reactions using primers (5′-GGAGAAGTGTGGGCTCCTC-3′ and 5′-AATCAAGGTCCGCTGTGAAC-3′) specific for the miR124–3 regulatory region.

### Imaging

Images were acquired on a Zeiss Axiovert 200 M fluorescent microscope using a × 63 oil-immersion objective (N.A. 1.4; ref. 62). Immunostained coverslips were mounted onto slides by using Prolong Gold anti-fade reagent and kept in the dark for 4 h before imaging. A DIC snap was first taken for morphology purposes. The exposure time for the fluorescence signal was first set automatically by the software and adjusted manually so that the signals were within the full dynamic range. Either the glow scale look-up table or the histogram was used to monitor the saturation level. Once the parameters were set, they were fixed and used throughout the imaging for the full set of experiments. Usually, —three to five sections of proximal dendrites per cell in 10–20 cells were used for analysis. AMPAR clusters were individually inspected to avoid contamination with nonspecific signals.

### Patch-clamp recordings

mEPSC recordings were performed as described previously^63^,^64^. Eleven-day-old cultured hippocampal neurons were transfected with plasmids as indicated and then supplemented with TTX (1 μM, Tocris Bioscience)/APV (50 μM, Tocris Bioscience) 48 h later for 15 h to induce homeostatic regulation. The coverslip was then transferred to a recording chamber with an extracellular solution containing (in mM) 140 NaCl, 3 KCl, 1.5 MgCl_2_, 2.5 CaCl_2_, 11 glucose and 10 HEPES (pH 7.4), which was supplemented with TTX (1 μM) to block action potentials, APV (50 μM) to block NMDAR and bicuculline (Sigma Aldrich, 20 μM) to block GABA_A_ receptor-mediated IPSCs. Cells that were co-expressing fluorescent protein were visually identified with a Zeiss Axiovert 200 M fluorescent microscope before recording. Whole-cell voltage-clamp recordings were made with patch pipettes pulled to an average resistance of 2–4 MΩ and filled with an intracellular solution containing (in mM) 100 Cs-methanesulfonate, 10 CsCl, 10 HEPES, 0.2 EGTA, 4 Mg-ATP, 0.3 Na-GTP and 10 Na-phosphocreatine (pH 7.4), with the membrane potential clamped at −70 mV with an average access resistance of 10–15 MΩ. Recordings started 10 min after establishing whole-cell configuration to ensure equilibration between the pipette solution and the cytosol. mEPSCs were recorded with an Axopatch 200B amplifier, displayed and recorded digitally on a computer for subsequent off-line analysis by Clampfit (pClamp version 10). For I–V curves, recordings were made from the same cell during a series of voltage steps. In some experiments, earlier time points were recorded to examine facilitated expression of the homeostatic response.

## Additional information

**How to cite this article:** Hou, Q. *et al*. MicroRNA miR124 is required for the expression of homeostatic synaptic plasticity. *Nat. Commun.* 6:10045 doi: 10.1038/ncomms10045 (2015).

## Supplementary Material

Supplementary Information Supplementary Figures 1-16 and Supplementary Tables 1-2
