# Supplementary material for: MicroRNA miR124 is required for the expression of homeostatic synaptic plasticity
Source: Nat Commun. 2015 Dec 1;6:10045. doi: 10.1038/ncomms10045 (PMC4686673; doi:10.1038/ncomms10045)
Supplement: Supplementary Information — Supplementary Figures 1-16 and Supplementary Tables 1-2 [file ncomms10045-s1.pdf]

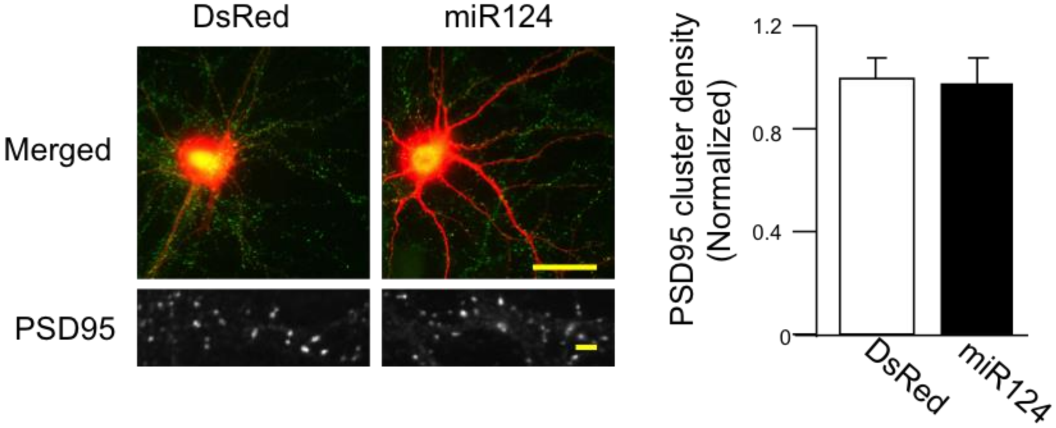


**Supplementary Figure 1. miR124 does not change neuron morphology and synaptic density.** Hippocampal neurons were transfected with miR124 (containing DsRed) or DsRed as a control. 2 d after transfection, neurons were immunostained for PSD95 (green). Expression of miR124 did not cause marked changes in neurite structure and density of PSD95 clusters. Bar graph represents Mean ± SE, n = 16 cells, $p>0.05$, t test. Scale bars = 10 μm (full images), 3 μm (dendrites).

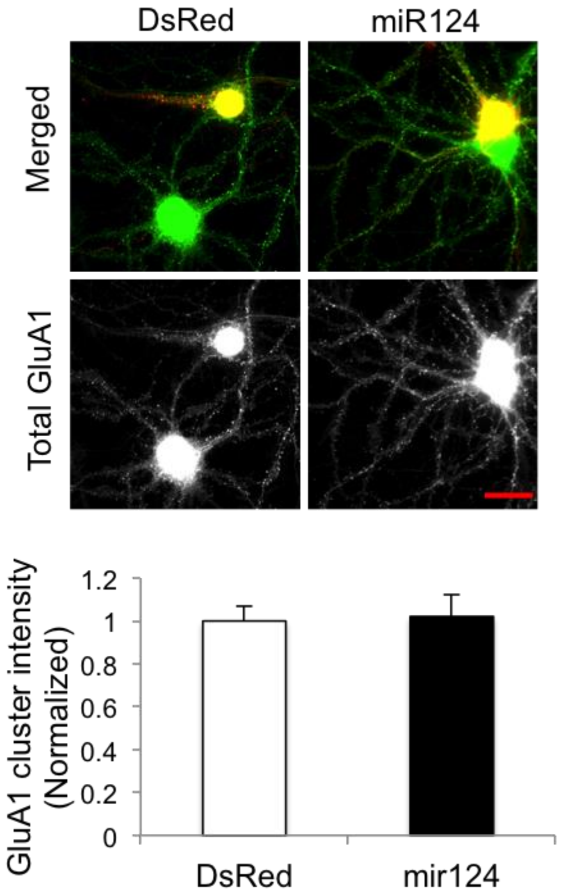


**Supplementary Figure 2. miR124 does not affect GluA1 expression.** Hippocampal neurons were transfected with miR124 (containing DsRed) or DsRed as a control. 2 d after transfection, neurons were immunostained for GluA1 (green) under permeant conditions. No changes in GluA1 cluster intensity were found in cells expressing miR124. Bar graphs represent Mean ± SE. n = 18 cells, t test. Image scale bar = 10 μm.

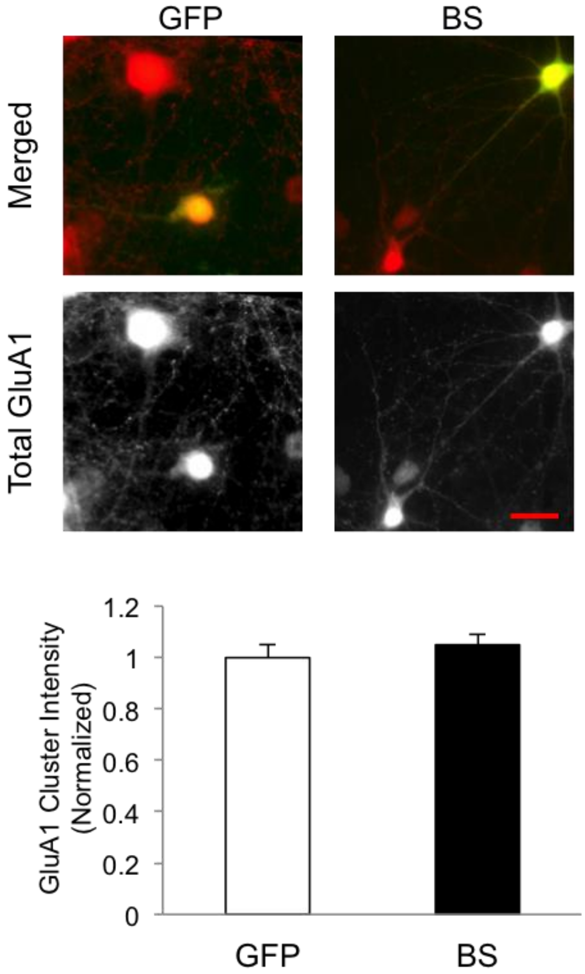


**Supplementary Figure 3. miR124 BS does not affect total GluA1 expression.** Cultured hippocampal neurons were transfected with a miR124-neutralizing sponge BS (containing GFP) at DIV12 and total GluA1 was immunostained 2 d later. GFP was transfected as a control. In cells expressing miR124 BS, the total GluA1 puncta intensity was not affected. Bar graph represents Mean ± SE, n = 10 cells, p>0.05, t test. Image scale bar = 10 μm.

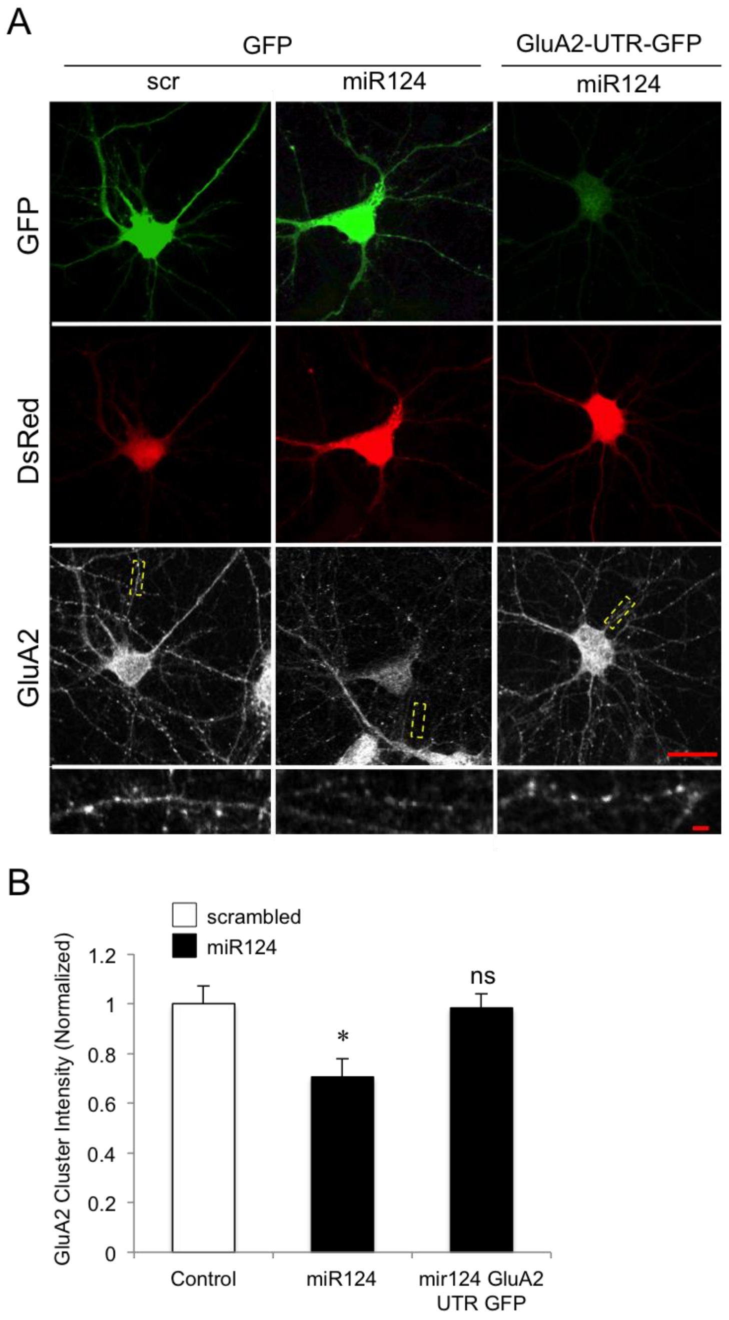


**Supplementary Figure 4. GluA2-UTR-GFP blocks the miR124-mediated decrease in GluA2 expression.** (A) DIV12 cultured hippocampal neurons were co-transfected with miR124 or a scrambled control (both contain DsRed) with GFP, or co-transfected with GluA2-UTR-GFP with miR124. Total GluA2 was immunostained 2 d later under permeant conditions.

(B) In cells expressing miR124, GluA2 levels were decreased compared to the scrambled control. However, miR124 did not change GluA2 in neurons expressing GluA2-UTR-GFP. Bar graph represents Mean ± SE, n = 9 - 10 cells, * = $p<0.05$, ns = $p>0.05$, t test. Image scale bars = 10 μm (full images), 3 μm (dendrites).

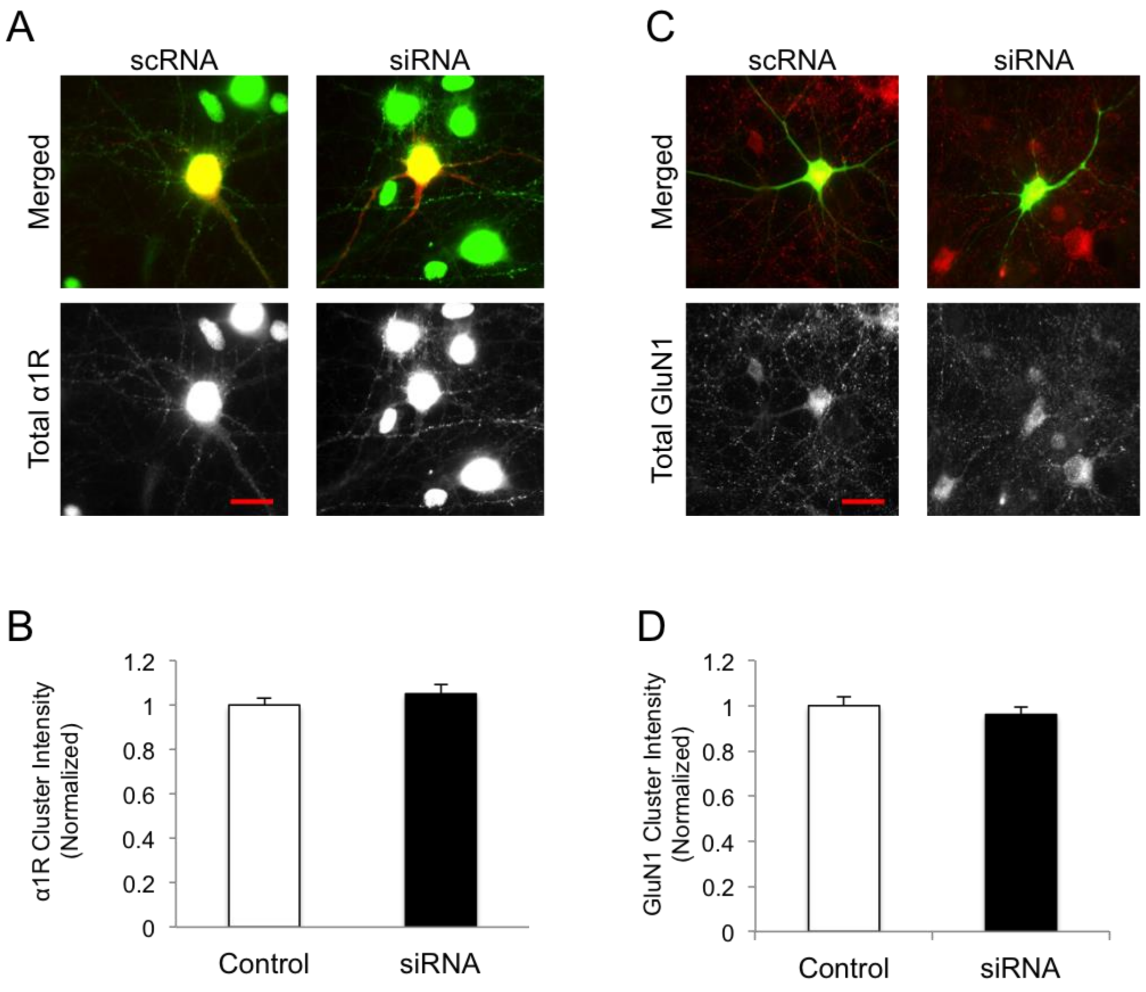


**Supplementary Figure 5. Suppression of miR124 does not affect GABAR and NMDAR expression.** Hippocampal neurons were transfected with siRNA against miR124, together with DsRed. Scrambled siRNA (scRNA) was used as a control. GABA receptor subunit α1 (α1R) or NMDA receptor subunit GluN1 were immunostained 2 d after transfection. Inhibition of miR124 did not affect the expression of α1R (A and B; n = 10 cells, $p>0.05$, t test) or GluN1 (C and D; n = 11 cells, $p>0.05$, t test). Image scale bars = 10 μm.

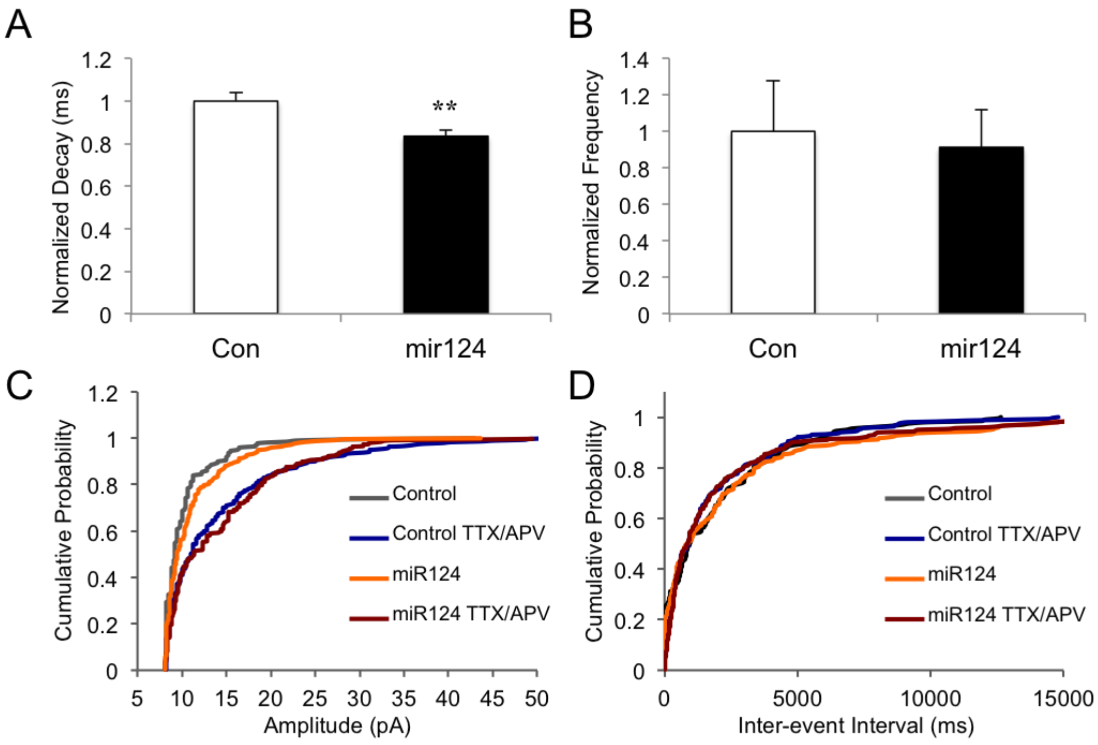


**Supplementary Figure 6. miR124-transfected cells show decreased mEPSC decay time and typical multiplicative homeostatic scaling.** (A) miR124 expressing cells showed a decreased in average mEPSC decay time. Bar graph represents Mean ± SE, n = 6 cells, ** = p<0.01, t test. (B) miR124 expression did not change mEPSC frequency. Bar graph represents Mean ± SE, n = 6 cells, p>0.05, t test.

(C and D) Cumulative probability plots of amplitude (C) and inter-event interval (D) show typical multiplicative scaling by TTX/APV incubation in miR124-transfected cells.

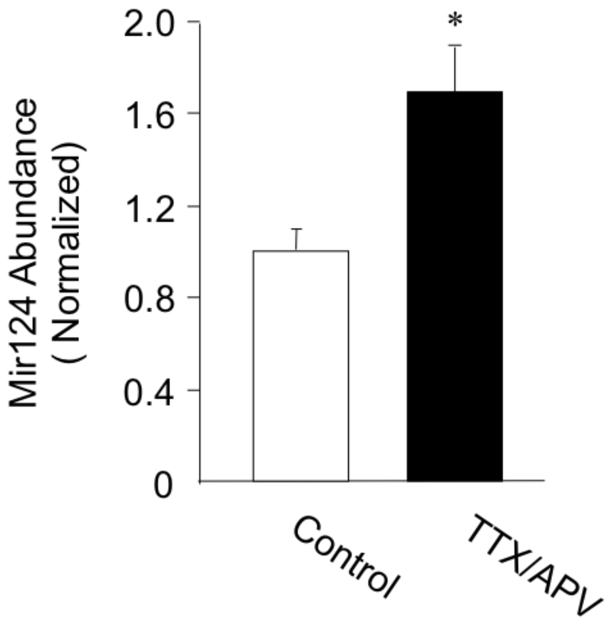


**Supplementary Figure 7. Neuronal inactivity causes an increase in mir124 expression.** Hippocampal neurons were incubated with TTX/APV for 15 hr. The amount of mir124 was measured by qPCR. Neuronal inhibition led to an increase in mir124. (n = 3, $p<0.05$, t test).

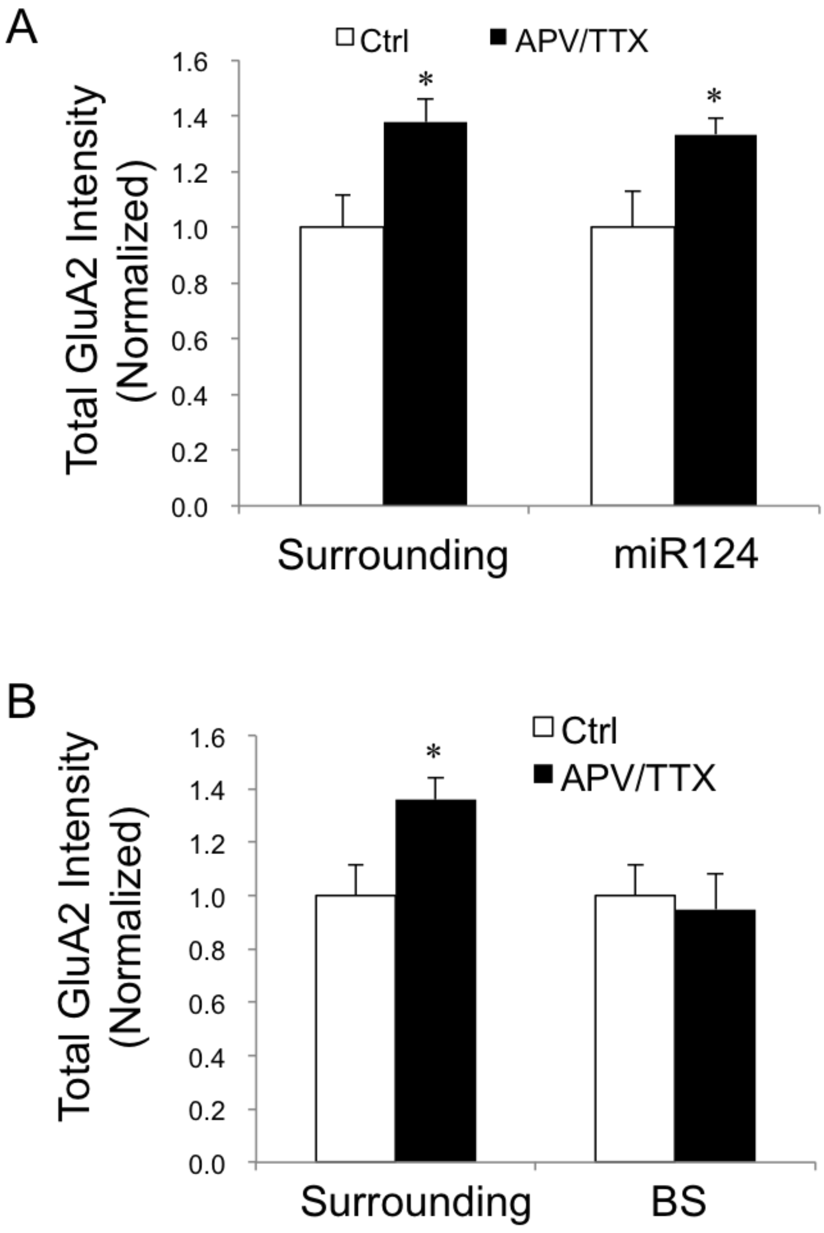


**Supplementary Figure 8. GluA2 is increased during HSP.** Neurons were transfected with mir124 or BS for 1 d, and then incubated with TTX/APV for 15 hr. Immunostaining showed a significant increase in GluA2 puncta intensity in both the non-transfected surrounding cells and the transfected cells (A). The homeostatic increase in GluA2 was blocked by BS (B).

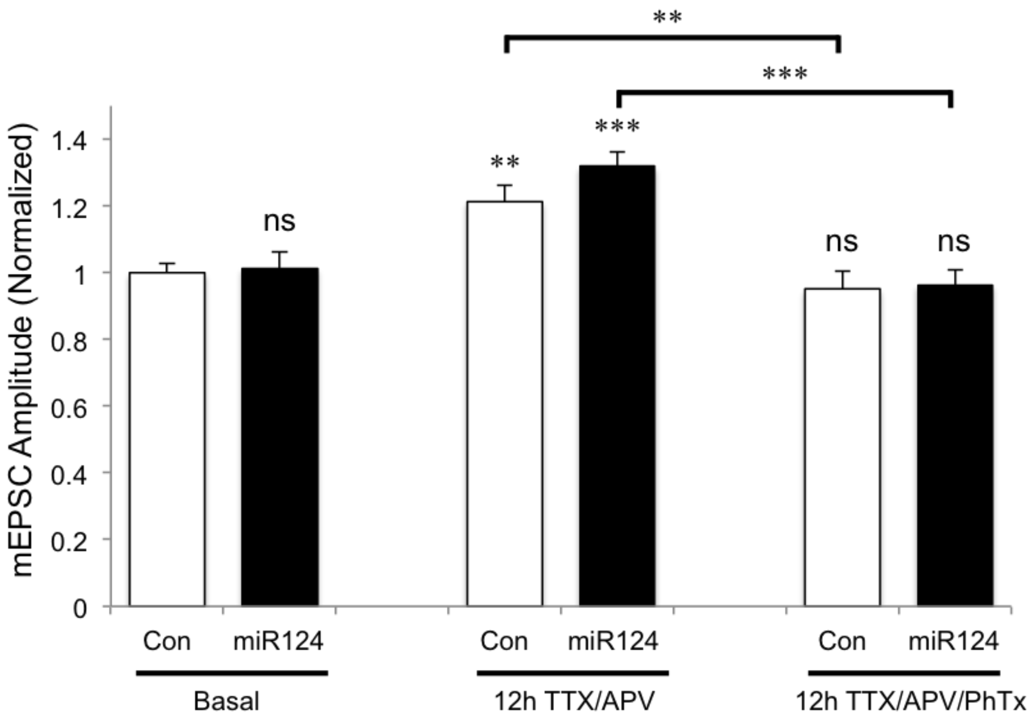


**Supplementary Figure 9. CP-AMPARs are required for HSP.** Hippocampal neurons were transfected with mir124 for 1 d, and then incubated with TTX/APV, with or without PhTx, for 12 hr. Application of PhTx abolished HSP expression shown by mEPSC recordings. (n = 5-8 cells, * = p<0.05, ** = p < 0.01, *** = p<0.001, ns = p>0.05, t test).

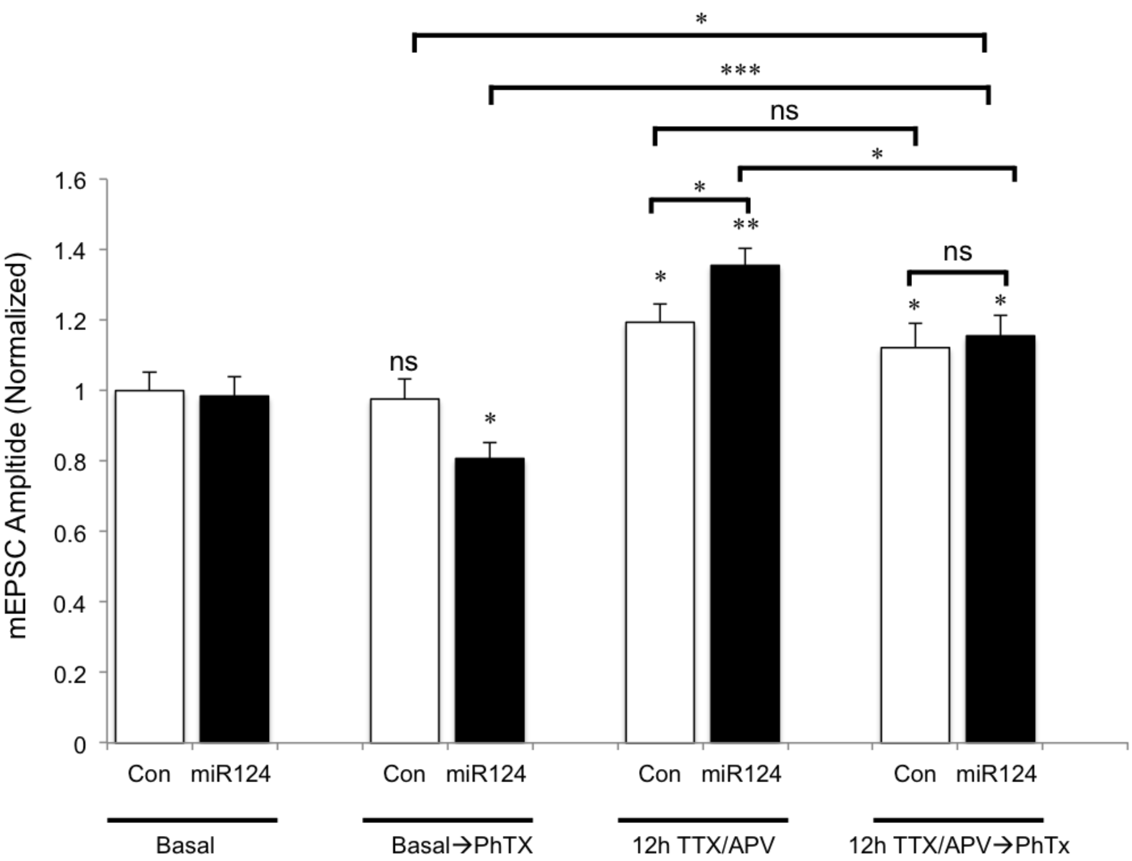


**Supplementary Figure 10. Contribution of GluA2-containing AMPARs in HSP.** Mir124-transfected neurons were incubated with TTX/APV for 12 hrs to induce HSP. Cells were then recorded in the absence and presence of PhTx. PhTx caused a decrease in mEPSC amplitude in mir124-transfected cells under control conditions (2nd pair of bar graphs). In TTX/APV treated cells, PhTx caused a partial decrease in mEPSC amplitude in mir124 transfected cells, but not in the control cells (3rd and 4th pairs of bar graphs) (n = 6-9 cells, * = p<0.05, ** = p<0.01, *** = p<0.001, ns = p>0.05, t test).

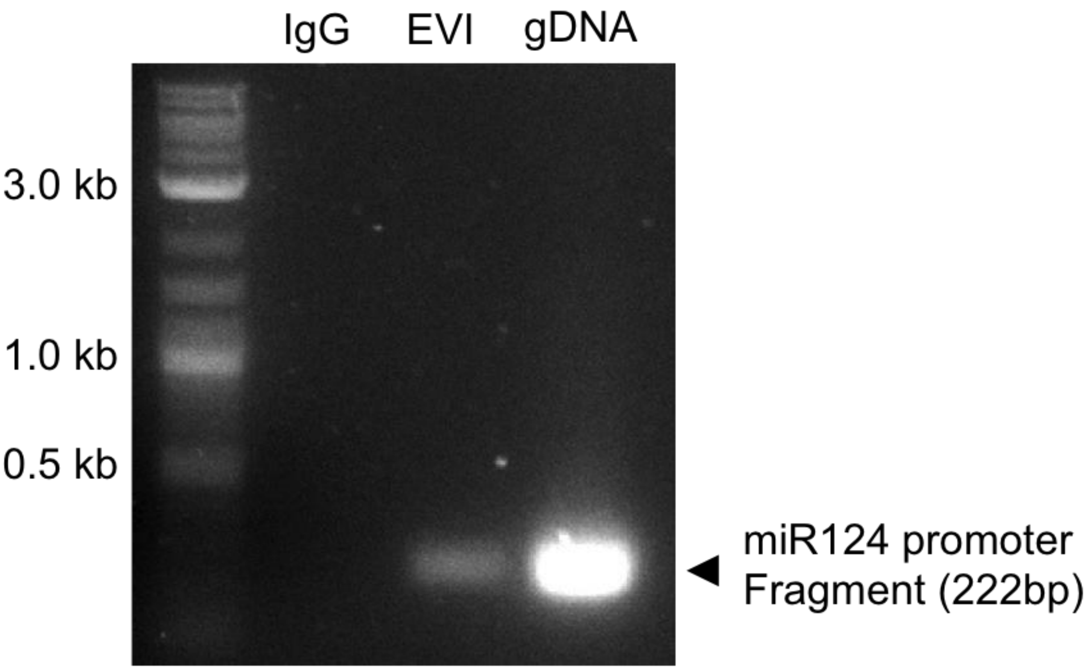


**Supplementary Figure 11. Association of EVI1 with mir124 promoter.** A ChIP assay shows positive detection of the miR124 promoter sequence in EVI1 immunoprecipitates from cortical neurons. Genomic DNA (gDNA) was used as control.

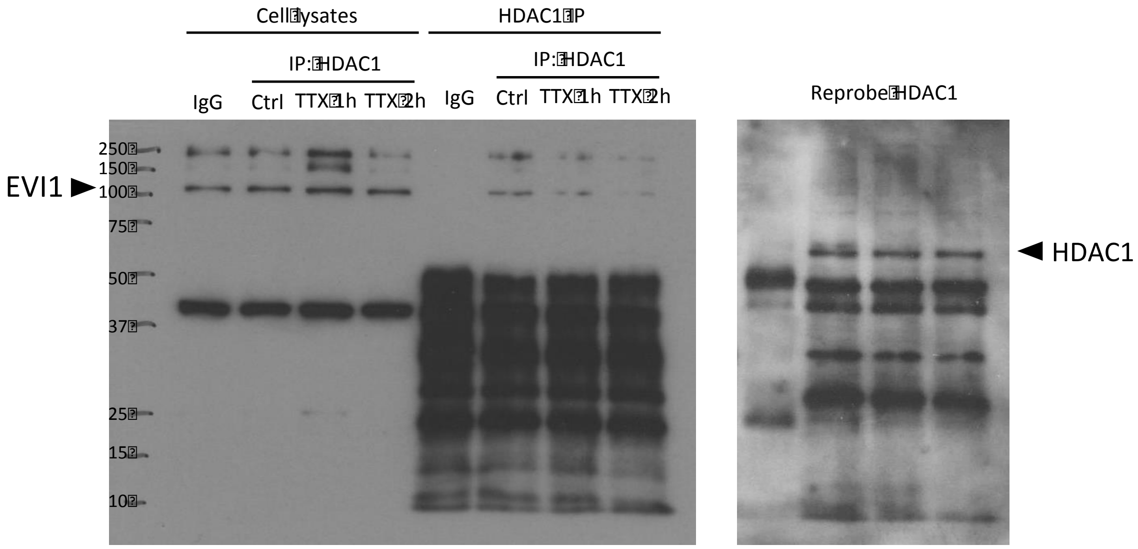


**Supplementary Figure 12. Co-IP of EVI1 and HDAC1.** Neurons were incubated with TTX/APV for 1 or 2 hr. Using the cell lysates, immunoprecipitates of anti-HDAC1 antibodies were probed for EVI1. HDAC1-EVI1 association was reduced by neuronal inhibition.

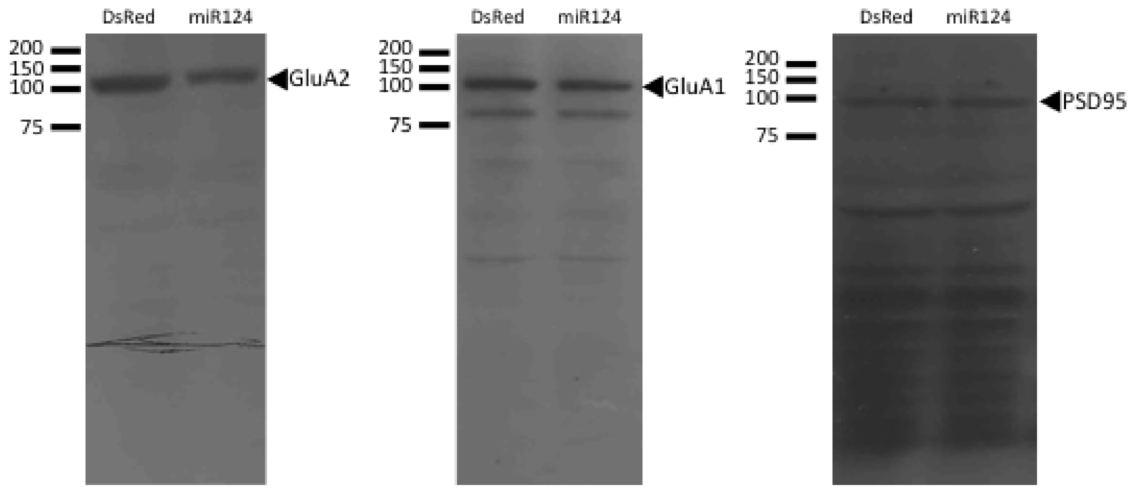


**Supplemental Figure 13.** Full western blots for Figure 1E.

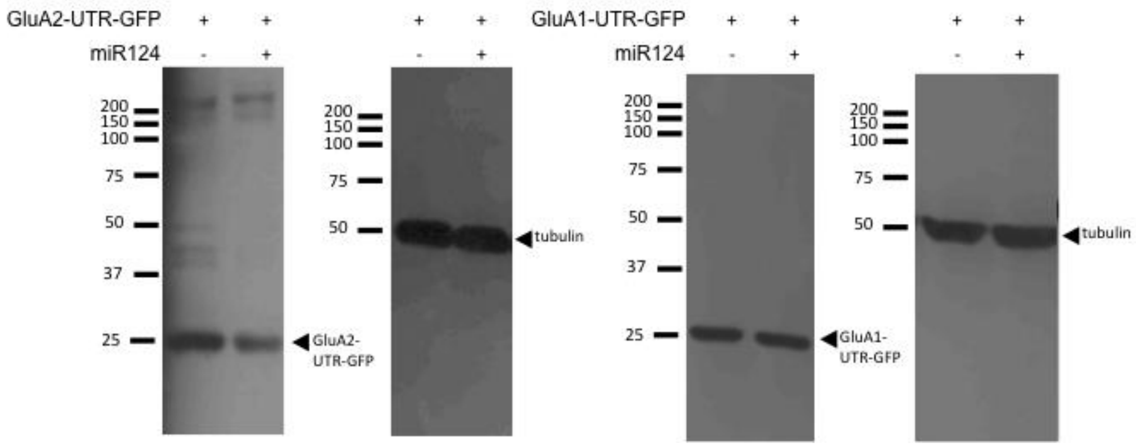


**Supplemental Figure 14.** Full western blots for Figure 2B.

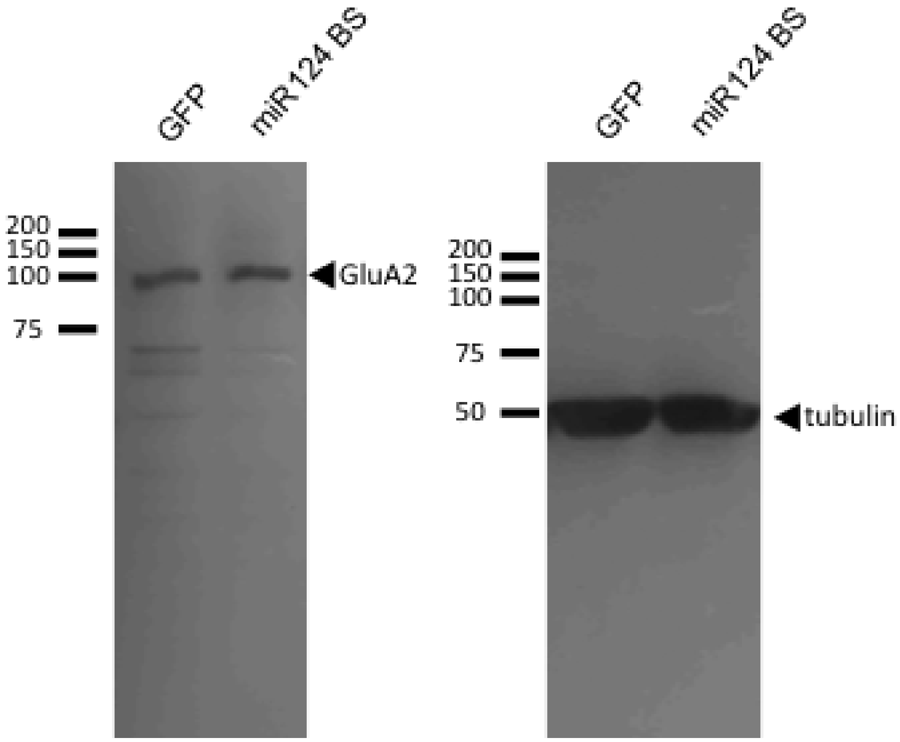


**Supplemental Figure 15.** Full western blots for Figure 3D.

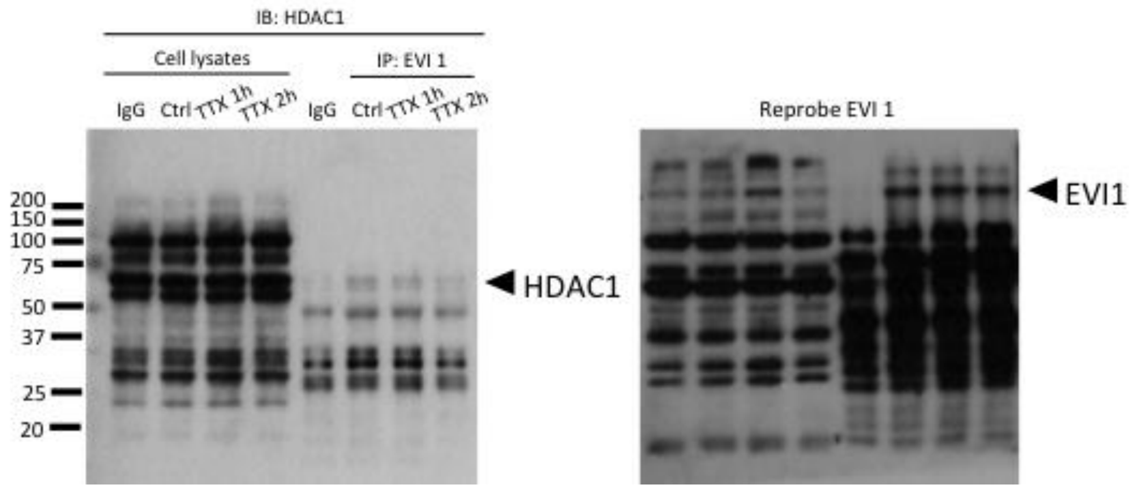


**Supplemental Figure 16.** Full western blots for Figure 7G.

| Treatment | Amplitude (pA) | SE |
|---|---|---|
| Con Basal | 12.77 | 0.37 |
| miR124 Basal | 12.95 | 0.62 |
| | | |
| Con TTX/APV | 15.48 | 0.65 |
| miR124 TTX/APV | 17.83 | 0.55 |
| | | |
| Con TTX/APV/PhTx | 12.15 | 0.66 |
| miR124 TTX/APV/PhTx | 12.30 | 0.60 |

**Table 1. mEPSC amplitudes from Supplementary Figure 9.**

| Treatment | Amplitude (pA) | SE |
|---|---|---|
| Con Basal | 13.82 | 0.48 |
| miR124 Basal | 13.84 | 0.78 |
| | | |
| Con Basal → PhTx | 13.74 | 0.79 |
| miR124 Basal → PhTx | 11.37 | 0.65 |
| | | |
| Con TTX/APV | 16.80 | 0.71 |
| miR124 TTX/APV | 19.08 | 0.68 |
| | | |
| Con TTX/APV → PhTx | 15.77 | 0.59 |
| miR124 TTX/APV → PhtX | 16.42 | 0.57 |

**Table 2. mEPSC amplitudes from Supplementary Figure 10.**
